# AJUBA: The Master Regulator Bridging EMT and Immune Evasion in Colorectal Cancer

**DOI:** 10.1155/mi/7828477

**Published:** 2026-03-11

**Authors:** Wenhui Shen, Minghui Cui, Xiaoqian Liao, Yuhan Xiong, Biji Zou, Xiaojun Zhang, Cuijie Shao

**Affiliations:** ^1^ Department of Head Neck and Thyroid, The Affiliated Cancer Hospital of Zhengzhou University and Henan Cancer Hospital, Zhengzhou, 450008, China, zzu.edu.cn; ^2^ Shandong First Medical University, Taian, 271000, China, sdfmu.edu.cn; ^3^ Clinical Laboratory, The Third Affiliated Hospital of Sun Yat-Sen University, Guangzhou, 510630, China, zssy.com.cn; ^4^ BASIS Bilingual School Shenzhen, Shenzhen, 518000, China; ^5^ Department of Medical Research Centor, Affiliated Hospital of Binzhou Medical University, Binzhou, China, bzmc.edu.cn

**Keywords:** AJUBA, colorectal cancer, overexpression, single-cell, spatial transcriptomics

## Abstract

**Background:**

Epithelial–mesenchymal transition (EMT) represents a critical process that facilitates metastatic dissemination and immune evasion in colorectal cancer (CRC); however, the molecular factors that connect EMT to modifications in the immune microenvironment remain poorly elucidated. In this investigation, we identify AJUBA as an essential regulator that mediates the association between EMT and immune modulation in CRC.

**Methods:**

By integrating multi‐cohort transcriptomic datasets (The Cancer Genome Atlas (TCGA)‐CRC, GSE18105, GSE22598, GSE89076, and GSE110224) with single‐cell RNA‐seq data (GSE132465), we applied machine learning and deep learning methodologies to comprehensively identify EMT‐associated genes demonstrating prognostic significance. AJUBA validation was performed at mRNA and protein levels in a cohort of 90 CRC patient samples using quantitative PCR, Western blotting, and immunohistochemical (IHC) approaches. Functional analyses involved siRNA‐mediated knockdown experiments, coupled with evaluations of cell proliferation (CCK‐8 assay), migration and invasion (transwell assay), clonogenic capacity (colony formation assay), and in vivo tumor growth in xenograft models. Immune infiltration was assessed via ssGSEA and CIBERSORT algorithms, and spatial transcriptomics data (GSE225857) were used to delineate AJUBA expression within tumor microdomains.

**Results:**

Across multiple CRC cohorts, AJUBA exhibited marked upregulation and showed distinct enrichment in epithelial cells with activated EMT characteristics. Spatial transcriptomic profiling demonstrated AJUBA colocalization with cancer‐associated fibroblasts (CAFs) within immune‐excluded niches. Enhanced AJUBA expression exhibited a positive correlation with heightened infiltration of M2 macrophages and activation of VEGF/NOTCH signaling cascades. In vivo, AJUBA knockdown led to suppressed tumor growth, reduced Ki‐67 proliferation indices, and diminished M2 macrophage abundance. Clinically, elevated AJUBA expression correlated with advanced nodal metastasis and served as an independent predictor of poor overall survival (OS; HR = 4.809, 95% CI: 2.385–9.695, *p* < 0.001).

**Conclusions:**

AJUBA functions as a key regulator that links EMT to immune modulation, promoting macrophage polarization and facilitating immune evasion in CRC. Through its coupling of EMT activation with proangiogenic signaling, AJUBA represents both a prognostic biomarker and a promising therapeutic target for alleviating immune exclusion in metastatic CRC.

## 1. Introduction

Colorectal cancer (CRC), a prevalent human malignancy, is characterized by uncontrolled proliferation of aberrant cells in the colon epithelium. It ranks as the third most commonly diagnosed cancer in Western countries. Dietary shifts are implicated in the 608,000 global deaths annually due to this disease [1]. Surgical procedures, including colectomy, constitute effective management for localized colon cancer at early stages. However, invasion of tumor cells into muscular tissues compromises the efficacy of surgical approaches, resulting in an unfavorable prognosis for advanced‐stage CRC.

The pathogenesis of CRC is centrally mediated by epithelial–mesenchymal transition (EMT), a fundamental biological process that facilitates tumor cell invasion, metastatic spread, and therapy resistance [2–4]. EMT molecular regulation involves a complex network consisting of transcription factors, signaling pathways, and extracellular stimuli, which collectively drive the shift from epithelial to mesenchymal phenotypes [2–4]. Advances in high‐throughput sequencing and single‐cell transcriptomics have renewed focus on identifying EMT‐associated genes that amplify tumor heterogeneity and promote aggressive disease progression [5–7]. However, the functional significance of numerous EMT‐linked genes remains inadequately clarified, and their prognostic relevance in CRC is not fully characterized. To systematically identify EMT‐related genes with potential clinical importance, we integrated multi‐cohort transcriptomic datasets—including TCGA‐CRC and four GEO repositories—applying conventional machine learning and deep learning methods. This integrated strategy enabled the classification of key EMT genes that display expression changes in CRC and carry substantial prognostic weight. Among these, AJUBA was consistently identified as the most prominently overexpressed gene, demonstrating strong prognostic associations across all training and validation cohorts.

AJUBA is classified as an actin‐binding protein that belongs to the LIM domain family. This protein family comprises AJUBA, LIMD1, WTIP, LPP, Zyxin, and Trip6 [7]. From a structural perspective, these proteins feature two or three successive LIM domains at the C‐terminus, combined with a characteristic preLIM domain at the N‐terminus [8]. In mammalian cells, AJUBA LIM proteins function as cytoplasmic adaptors that connect cell adhesion signaling to nuclear functions, thereby promoting epithelial remodeling [9]. Studies have shown that AJUBA increases cell proliferation by suppressing the Hippo/YAP signaling pathway [10]. Accumulating evidence connects AJUBA to tumorigenesis, owing to mutations identified in multiple human cancers, such as cutaneous squamous cell carcinoma [5], head and neck squamous cell carcinomas [6], and esophageal squamous cell carcinoma [7, 11–13]. Additionally, various studies indicate that AJUBA family proteins may operate as potential tumor suppressors [11, 12, 14, 15].

This investigation identifies AJUBA as a pivotal regulator linked to EMT in CRC. Through integration of bulk RNA‐seq, single‐cell RNA‐seq, and experimental analyses in clinical samples, we defined the expression patterns, functional characteristics, and prognostic relevance of AJUBA in CRC. Our data present initial comprehensive evidence demonstrating that AJUBA functions both as an EMT‐enriched gene and an independent prognostic biomarker in CRC, emphasizing its therapeutic potential within precision oncology.

## 2. Materials and Methods

### 2.1. Patient Characteristics and Tissue Repository

This investigation utilized a cohort of 90 formalin‐fixed, paraffin‐embedded (FFPE) colon adenocarcinoma specimens, along with matched normal adjacent tissues obtained from sites at least 5 cm from the tumor margin. All samples were acquired from Binzhou Medical University Hospital between July 2006 and May 2007 and were subjected to immunohistochemical (IHC) analysis. Enrolled patients had a median age of 70 years (range: 24–90 years), and a median tumor diameter of 5.5 cm (range: 1.2–15.0 cm). Each participant received a pathological diagnosis of colon adenocarcinoma, had no prior neoadjuvant treatment, and was treated with curative surgical resection. Clinical characteristics of the cohort are summarized in Table 1. For survival analysis, the date of surgery was set as the baseline (time zero), and overall survival (OS) was calculated as the duration from surgical intervention to death due to any cause.

**Table 1:** Association between AJUBA expression and clinicopathologic characteristics.

Characteristics	Total (*n* = 90)	AJUBA		*p*‐Value
		Negative (*n* = 40)	Positive (*n* = 50)	
Gender				0.186
Male	47 (52.2%)	24 (51.1%)	23 (50.7%)	
Female	43 (47.8%)	16 (37.2%)	27 (48.9%)	
Age (years)				0.185
≥60	73 (81.1%)	30 (41.1%)	43 (58.9%)	
＜60	17 (18.9%)	10 (58.8%)	7 (41.2%)	
T stage				0.292
1	4 (4.4%)	3 (75.0%)	1 (25.0%)	
2	7 (7.8%)	2 (28.6%)	5 (71.4%)	
3	68 (75.6%)	32 (47.1%)	36 (52.9%)	
4	11 (12.2%)	3 (27.3%)	8 (72.7%)	
N stage				0.001
0	56 (62.2%)	36 (64.3%)	20 (35.7%)	
1	25 (27.8%)	4 (16.0%)	21 (84.0%)	
2	9 (10.0%)	0 (0.0%)	9 (100.0%)	
M stage				0.201
0	88 (97.8%)	40 (45.5%)	48 (54.5%)	
1	2 (2.2%)	0 (0.0%)	2 (100.0%)	
TNM stage				0.001
Ⅰ	7 (7.8%)	4 (57.1%)	3 (42.9%)	
Ⅱ	48 (53.3%)	32 (66.7%)	16 (33.3%)	
Ⅲ	33 (36.7%)	4 (12.1%)	29 (87.9%)	
IV	2 (2.2%)	0 (0.0%)	2 (100.0%)	
Tumor size (cm)				0.811
≥5	53 (58.9%)	23 (43.4%)	30 (56.6%)	
<5	37 (41.1%)	17 (45.9%)	20 (54.1%)	
Grade				0.425
1	5 (3.8%)	1 (20.0%)	4 (80.0%)	
2	49 (23.6%)	21 (42.9%)	28 (57.1%)	
3	36 (71.7%)	18 (50.0%)	18 (50.0%)	
Infiltration				0.115
0	87 (96.7%)	40 (46.0%)	47 (54.0%)	
1	3 (3.3%)	0 (0.0%)	3 (100.0%)	

This investigation incorporated 10 matched pairs of colon carcinoma and adjacent normal tissue specimens (applying equivalent standards for normal adjacent tissue delineation) sourced from Binzhou Medical College Hospital from January through June 2015 for quantitative real‐time PCR (RT–PCR) evaluation. An independent group of 10 paired samples, including cancerous and matched normal colonic tissues, was obtained from the same facility between February 2016 and May 2018 for Western blot assessment. All specimens were secured shortly after surgical removal.

Clinical and pathological classification and staging were executed in accordance with the criteria specified in the Seventh Edition of the American Joint Committee on Cancer (AJCC) guidelines. Ethical clearance for this research was issued by the Ethics Committee of Binzhou Medical College, and written informed consent was acquired from all participants to authorize the utilization of clinical specimens within this study.

### 2.2. Data Collection and Preparation

The comprehensive bioinformatics pipeline employed in this study was adapted from established protocols for multi‐omics analysis of cancer biomarkers [16]. This investigation utilized data sourced from The Cancer Genome Atlas (TCGA) and the Gene Expression Omnibus (GEO), including the TCGA‐CRC, GSE18105, GSE22598, GSE89076, and GSE110224 datasets. Subsequent analysis entailed the conversion of level 3 HTSeq‐FPKM (RNA‐seq) data from the TCGA‐CRC cohort into transcripts per million (TPM) units [17, 18]. Differential gene expression between colorectal carcinoma samples and normal controls was evaluated using the ‘limma’ package [19] within the R statistical framework [20, 21]. Selection criteria for upregulated differentially expressed genes (DEGs) mandated a log_2_ fold change greater than 1.5 and an adjusted *p*‐value below 0.05, while downregulated DEGs were defined by a log_2_FC lower than −1.5 with an adjusted *p*‐value under 0.05 [22]. Furthermore, 1184 EMT‐associated genes were acquired from the dbEMT2 repository (https://bioinfo-minzhao.org/dbemt/index.html) [23].

### 2.3. A Complete Workflow for scRNA‐Seq Data Analysis

We conducted a comprehensive analysis of the single‐cell RNA sequencing dataset GSE132465 from CRC. In scRNA‐seq quality control, cells meeting predetermined exclusion criteria were eliminated, including those containing fewer than 200 detected genes, those exhibiting over 5000 genes (suggestive of doublets), and those where mitochondrial gene proportions surpassed 10% of total counts [24]. Normalization, dimensionality reduction, and clustering of data were executed with the Seurat package [25]. To counteract batch effects that could compromise analytical reliability, the top 2000 highly variable genes were corrected using the Harmony package under default parameters. Systematic assessment identified a resolution of 0.5 as optimal for visualizing clusters. Cell type annotation was carried out by manually consulting colorectal‐specific marker genes in the ACT database [26] (http://xteam.xbio.top/ACT/index.jsp), integrated with automated analysis via the UCell algorithm utilizing cell‐type signature gene sets [27].

### 2.4. Machine Learning and Deep Learning Algorithms Screen Genes

The TCGA‐CRC cohort served as the primary discovery (training) dataset, while independent GEO series—GSE18105, GSE22598, GSE89076, and GSE110224—were designated as external validation sets. Feature selection within the training dataset was conducted using a 10‐fold cross‐validation strategy. An extensive assessment was performed incorporating over 10 computational methodologies, evaluated both individually and in diverse ensemble and hybrid configurations [28]. The methods assessed included logistic regression (LR), linear discriminant analysis (LDA), quadratic discriminant analysis (QDA), k‐nearest neighbors (KNN), decision tree (DT), random forest (RF), extreme gradient boosting (XGBoost), ridge regression, Lasso, Elastic Net, support vector machine (SVM), gradient boosting machine (GBM), stepwise selection based on Akaike’s information criterion (StepWise‐AIC), and Naive Bayes. Using TensorFlow [29], we developed a deep learning architecture to identify genes associated with EMT in the TCGA‐CRC dataset. Gene expression data derived from the GSE18105, GSE22598, GSE89076, and GSE110224 datasets were uniformly processed and normalized relative to the TCGA‐CRC cohort for model construction. Optimization of the model required careful tuning of multiple hyperparameters: batch sizes of 5 and 10, learning rates set at 0.001, 0.005, and 0.010, alongside dropout rates of 0.25 and 0.50. Classification thresholds were established at 0.25, 0.50, and 0.75, and neural network architectures comprised layers with 32, 16, 8, and 1 neuronal units. L2 regularization was applied using *λ* values of 0 and 0.001, with training conducted over 50 epochs. For model evaluation, SHAP analysis determined gene‐level contributions to CRC, loss and performance measures were systematically documented, and ROC curves examined predictive accuracy in CRC [27].

### 2.5. RT–PCR Analysis

Total RNA was extracted from primary colon tumor samples and matched adjacent normal tissues employing Trizol reagent (Invitrogen, CA, USA) according to the manufacturer’s guidelines. After purification, the RNA received DNase treatment to eliminate genomic DNA contamination. Synthesis of cDNA was achieved through reverse transcription with 2 μg of RNA per sample. Amplification of Ajuba cDNA was performed using these parameters: initial denaturation at 95°C for 10 min; 36 cycles consisting of denaturation at 95°C for 20 s, annealing at 56°C for 20 s, and extension at 72°C for 20 s; concluding with a final extension at 72°C for 5 min. The amplified products were then maintained at 4°C. Relative Ajuba mRNA expression in tumor tissues versus normal tissues from the same individuals was evaluated via quantitative RT–PCR. The primer sequences employed were: Ajuba forward, 5′‐ATGGGGAAGTCCTATCATCCAG‐3′; Ajuba reverse, 5′‐TGGTAGTCGGTGACACAGTAT‐3′. GAPDH functioned as the internal control with primers: forward, 5′‐TGTTGCCATCAATGACCCC‐3′; reverse, 5′‐CTCCACGACGTACTCAGC‐3′. All primers were constructed using Primer Express v2.0 software (Applied Biosystems). Every experimental procedure was conducted in triplicate to verify reproducibility [30].

### 2.6. Western Blotting Analysis

Tumor and matched normal tissue specimens were initially pulverized in liquid nitrogen and subsequently lysed in SDS‐PAGE sample buffer. Protein separation was conducted using 10.5% SDS‐polyacrylamide gels, followed by electrophoretic transfer onto PVDF membranes (Immobilon P; Millipore, Billerica, MA, USA). Membranes were saturated by incubation with 5% nonfat milk dissolved in TBST (Tris‐buffered saline containing 0.1% Tween 20) for 1 h at room temperature. After blocking, membranes were treated overnight at 4°C with an anti‐Ajuba antibody (1:1000, 15865‐1‐AP; Proteintech Group, Inc., Rosemont, IL, USA), and subsequently with horseradish peroxidase‐conjugated goat anti‐rabbit IgG (SC 2004; Santa Cruz Biotechnology, Inc.). Ajuba protein expression was visualized by employing ECL Western blotting detection reagent (Amersham/GE Healthcare Life Sciences) in accordance with the manufacturer’s guidelines. GAPDH (1:5,000; Proteintech Group, Inc.) served as the internal loading control [31].

### 2.7. IHC Analysis

IHC analysis was utilized to assess protein expression changes in 90 human colon carcinoma specimens along with 90 matched adjacent nontumorous tissues. Briefly, 4‐μm‐thick sections from paraffin‐embedded tissue blocks underwent deparaffinization in xylene and sequential rehydration. Antigen retrieval was performed by microwave heating of slides in EDTA buffer. Endogenous peroxidase activity was quenched by treatment with 3% hydrogen peroxide in methanol, and nonspecific binding was blocked using 1% bovine serum albumin. Tissue sections were incubated overnight at 4°C with a rabbit polyclonal anti‐AJUBA antibody (1:100; Proteintech, Catalog number: 10653‐1‐AP). Normal goat serum served as the negative control. After three washes, sections were treated with a biotinylated anti‐rabbit secondary antibody (Abcam), followed by incubation with a streptavidin–horseradish peroxidase complex (ZLI‐9017, Beijing Zhongshan Biotechnology Co., Ltd.; Beijing, China). Signal development was carried out using 3‐amino‐9‐ethyl carbazole, followed by counterstaining with 10% Mayer’s hematoxylin, dehydration, and mounting with Crystal Mount [32].

Two pathologists, remaining blinded to all histopathological and clinical details, conducted independent evaluations of the IHC staining. To ensure reliable quantification of AJUBA expression, composite scores integrating both staining intensity and the proportion of stained cells were calculated as the mean of the two evaluators’ independent assessments. Staining intensity was assessed on a scale ranging from 0 to 3, with 0 denoting no staining, 1 indicating faint yellow signal, 2 representing moderate yellow‐brown intensity, and 3 corresponding to intense brown staining. The proportion of positively stained tumor cells was scored according to a five‐tier system: 0 (0%), 1 (1%–25%), 2 (26%–50%), 3 (51%–75%), and 4 (>75%).

The staining index was calculated as the product of the percentage of positively stained tumor cells and the intensity score. AJUBA expression levels were stratified based on predefined criteria: a score of 0 was defined as negative (“−”), values between 1 and 4 were considered weakly positive (“+”), scores from 5 to 8 were designated positive (“++”), and those from 9 to 12 were classified as strongly positive (“+++”). Optimal thresholds for AJUBA expression were established by examining heterogeneity in OS using log‐rank testing. Elevated AJUBA expression was defined by a staining index ≥4, while indices below 4 indicated low expression.

### 2.8. Cell Culture and si‐RNA Transfection

The HCT116 and RKO cell lines were obtained from the American Type Culture Collection (ATCC) and maintained in RPMI‐1640 medium containing 10% fetal bovine serum (FBS; BI, Cromwell, CT, USA). For experiments, cells were seeded in six‐well plates and grown under serum‐free conditions, with medium changed at 70% confluence [33].

Gene knockdown was achieved by introducing 7.2 μL of AJUBA‐directed small interfering RNA (siRNA, 20 μM) using Lipofectamine 3000 reagent (Thermo Fisher Scientific) as specified by the manufacturer. The transfection mixture was applied dropwise to culture plates and swirled gently for uniform coverage; full medium was restored after 6 h. Analyses were performed 24–48 h post‐transfection. The siRNA constructs AJUBA‐1 and AJUBA‐2, with sequences si‐AJUBA‐1:5′‐AGCUAAACUGACUAGAACCAAAUCA‐3′ and si‐AJUBA‐2:5′‐ACUUCUGAGCUAUUAUCAGCAACAT‐3′, were synthesized by Gene Pharma (Shanghai, China). To evaluate individual siRNA effects on AL365181.3 expression and related phenotypes, each siRNA was used independently without combination.

### 2.9. CCK8, Colony Formation, and Transwell Migration and Invasion Assays

Cell proliferation was evaluated using the CCK‐8 assay by seeding cells in 96‐well plates at a density of 1 × 10^3^ cells/well and maintaining them for specified durations. Proliferation measurements were obtained with a CCK‐8 assay kit (Yeasen, Shanghai, China) following the manufacturer’s instructions. Colony‐forming ability was determined by culturing cells in 35‐mm dishes for 12 days; subsequently, colonies were fixed and stained with crystal violet solution to allow visualization and quantitative evaluation.

Cell suspensions were prepared in serum‐free medium. For invasion studies, Transwell inserts were precoated with Matrigel (BD Biosciences, San Jose, CA, USA). The upper chamber was filled with 100 μL of serum‐free medium containing cell suspensions (1 × 10^5^ cells per well for invasion or 4 × 10^4^ for migration assays), while the lower chamber contained 600 μL of medium supplemented with 20% FBS. After incubation, noninvading cells on the upper membrane surface were removed by PBS washing and fixed with paraformaldehyde. Cells that had migrated to the lower side of the membrane were stained with crystal violet and observed using an inverted light microscope (Leica Microsystems, Buffalo Grove, IL, USA).

### 2.10. Xenograft Model Studies

Four‐week‐old female BALB/c nude mice were obtained from GemPharmatech Technology (Jiangsu, China). HCT116 cells (5 × 10^6^) were injected subcutaneously into the left flank region of each mouse. Animals were allocated into experimental cohorts of six, with each mouse receiving six doses of either si‐NC or chemically modified siRNA (20 nmol, 2′‐OMe‐modified) every 48 h in 0.1 mL saline buffer. On Day 13, when tumors reached dimensions of ~5 mm × 5 mm, xenograft models were randomly divided into two treatment groups (*n* = 3 per group). At Day 29, tumor‐bearing mice were anesthetized with isoflurane and euthanized by cervical dislocation. Tumors were subsequently excised, weighed, and photographed for documentation. Animal procedures followed the ARRIVE 2.0 guidelines and were randomized; investigators were blinded during tumor measurement.

### 2.11. Statistical Analysis

OS was defined as the duration from randomization to death from any cause or the final follow‐up time for censored cases. Statistical analyses were performed with SPSS software (version 20.0). Differences in AJUBA expression between colorectal carcinoma tissues and matched noncancerous adjacent samples were analyzed using the chi‐square test. Survival outcomes were depicted through Kaplan–Meier curves, with statistical comparisons between groups conducted via the log‐rank test. Relationships between AJUBA expression and clinicopathological characteristics were investigated employing chi‐square and Fisher’s exact tests. Pairwise associations among clinicopathological parameters were evaluated by calculating Spearman’s rank correlation coefficients. Prognostic factors were identified through univariate and multivariate Cox regression analyses: univariate assessment utilized the enter method, while multivariate modeling applied the forward approach. A *p*‐value under 0.05 was considered statistically significant.

## 3. Results

### 3.1. Machine‐Learning and Deep‐Learning Identification of EMT‐Related Genes in CRC

Differential gene expression was analyzed across multiple CRC datasets, namely TCGA‐CRC, GSE18105, GSE22598, GSE89076, and GSE110224. Genes with |log fold change (logFC)| exceeding 1.5 and adjusted *p*‐values below 0.05 formed the basis of gene selection. Multigroup volcano plots presented in Figure 1A illustrate these findings. Subsequently, overlapping genes among upregulated candidates from the five datasets and 1184 EMT‐related genes obtained from the dbEMT2 database were subjected to intersection analysis. This combined approach led to the identification of 12 potential hub genes, as shown in Figure 1B.

Figure 1Identification of genes linked to epithelial–mesenchymal transition in colorectal cancer. (A) A volcano plot that combines data from multiple patient cohorts, illustrating the differences in gene expression levels within colorectal cancer RNA sequencing datasets. (B) A petal diagram showcasing the overlap among genes that are upregulated and those identified as epithelial–mesenchymal transition (EMT)‐related biomarkers across various colorectal cancer study groups. (C) A heatmap displaying the top 50 AUC values obtained from the application of diverse machine learning models to different subsets of patient data. Learning curve analysis for the neural network‐based multilayer perceptron model. (D) The pattern of loss fluctuation observed throughout the training process across multiple epochs. (E) The progression of accuracy metrics for both the training and validation datasets as the model undergoes optimization. (F) A diagram that ranks the importance of individual features as determined by the machine learning algorithm. (G) Scatter plots of SHAP (SHapley Additive exPlanations) values produced by the optimized multilayer perceptron neural network, highlighting the influence of specific EMT‐associated genes on the predictive performance for colorectal cancer.

**Figure:**
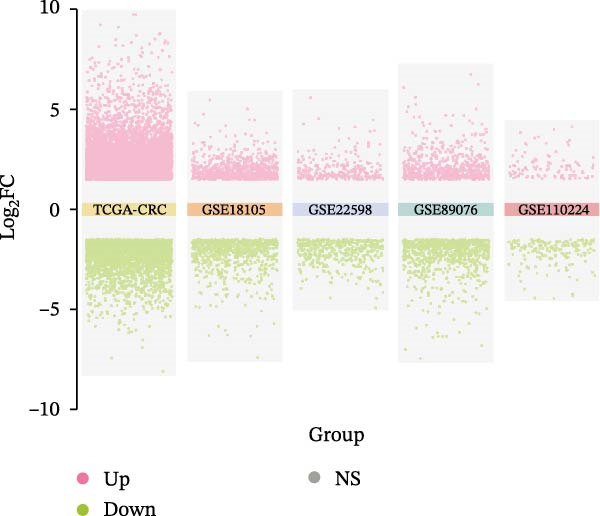
(A)

**Figure:**
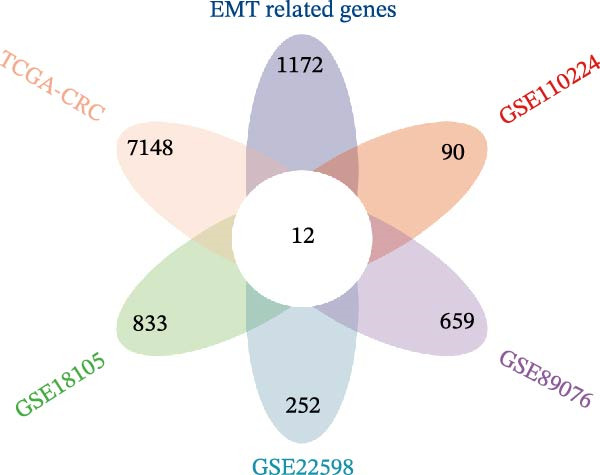
(B)

**Figure:**
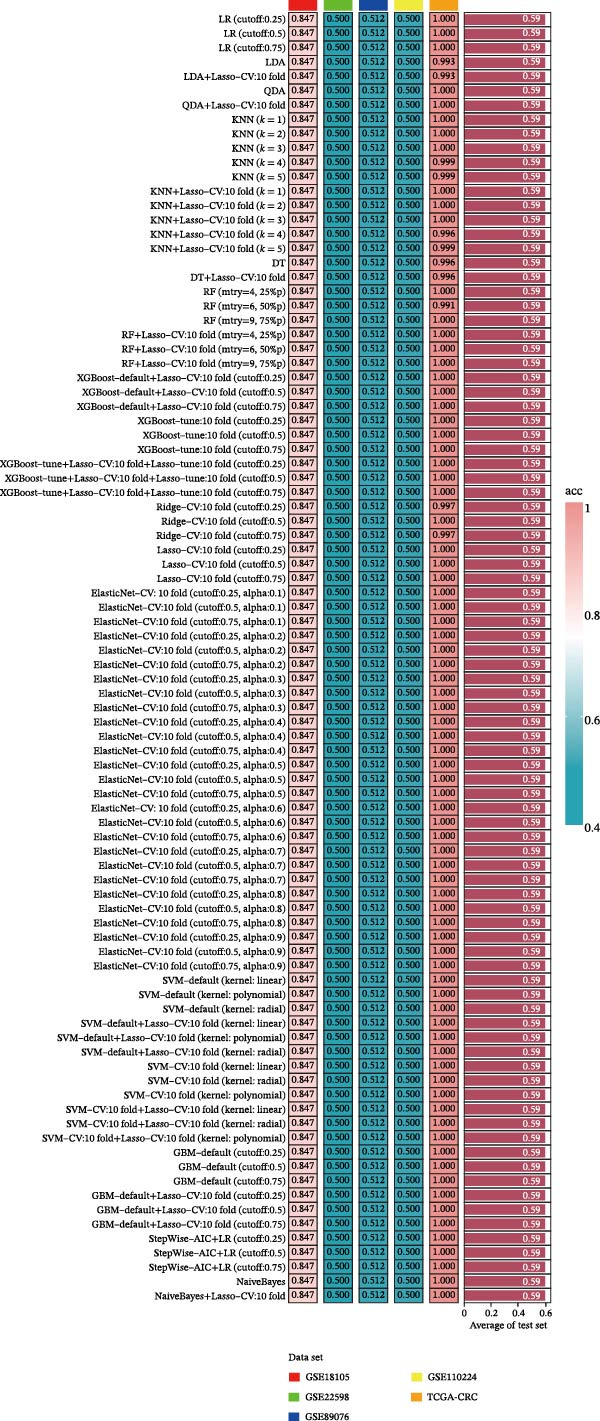
(C)

**Figure:**
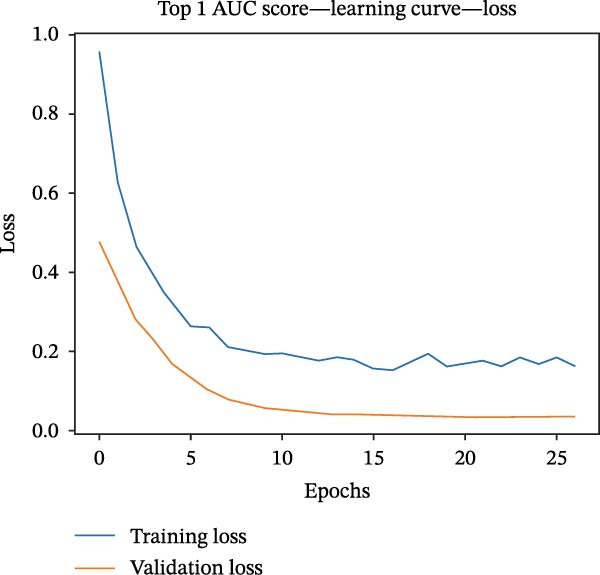
(D)

**Figure:**
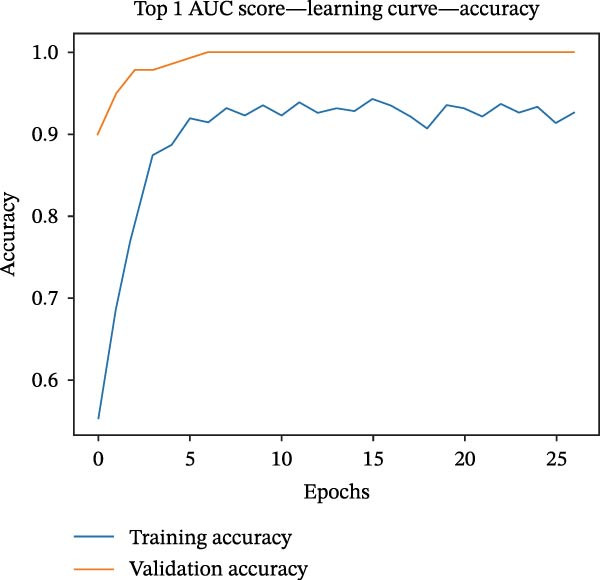
(E)

**Figure:**
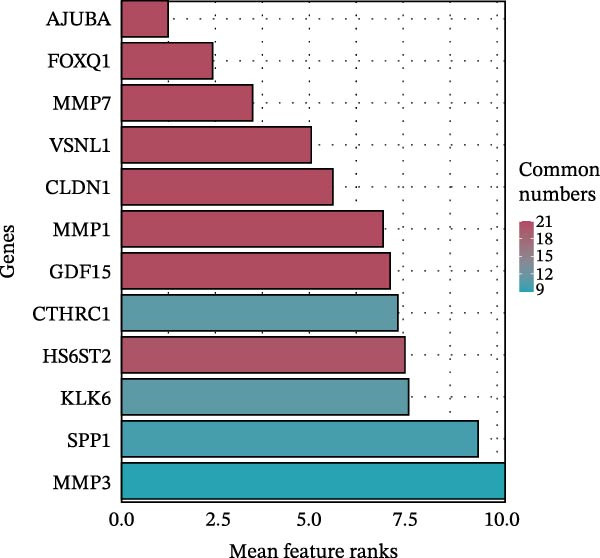
(F)

**Figure:**
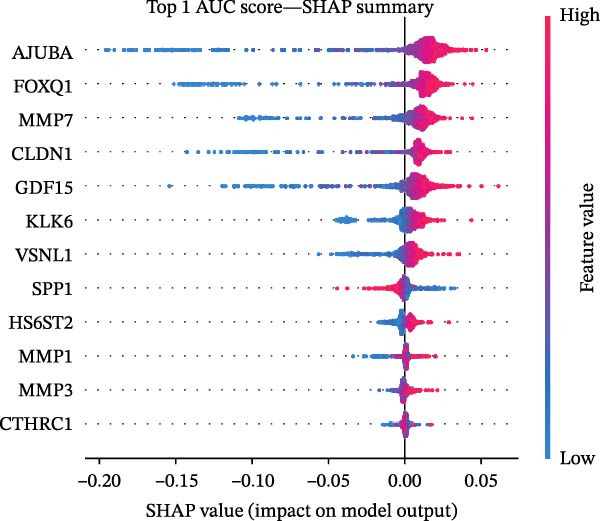
(G)

To refine gene selection, OmicsTools (https://github.com/zihaoxingstudy1/OmicsTools) was employed to assess various configurations encompassing over 10 machine learning algorithms and a dedicated deep learning approach termed the NN‐MLP algorithm. The TCGA‐CRC cohort functioned as the training dataset, with independent validation performed on GSE18105, GSE22598, GSE89076, and GSE110224. Throughout model development, a 10‐fold cross‐validation strategy was implemented to integrate multiple predictive frameworks, accompanied by evaluation of the accuracy curve area in training and validation sets (Figure 1C). Among models generated through diverse machine learning techniques, LR achieved the highest accuracy (cutoff: 0.25). Gene importance rankings are illustrated in Figure 1F. The NN‐MLP model was subsequently applied to evaluate the 12 candidate genes. Learning curves depicting loss and accuracy are shown in Figure 1D,E. Figure 1G exhibits a scatter plot of SHAP values derived from the trained multilayer perceptron model, emphasizing the role of pivotal genes in CRC prediction. Integrated results from machine and deep learning analyses consistently identified AJUBA as the highest‐ranked gene. Consequently, AJUBA was designated as the primary gene for further investigation.

### 3.2. Immune Infiltration Landscape and Macrophage Enrichment of AJUBA

Computational immunogenomic analyses were performed on bulk transcriptomic data from CRC (measured in TPM) employing ssGSEA and CIBERSORT approaches [34]. Spearman correlation revealed a significant positive relationship between AJUBA expression and total macrophage abundance (ssGSEA; Figure 2A). When tumors were stratified by median AJUBA expression, elevated macrophage infiltration scores were observed in the high‐AJUBA subgroup (Figure 2B). Consistently, CIBERSORT‐based cellular deconvolution indicated positive associations of AJUBA with M0, M1, and M2 macrophage subsets (Figure 2C). Comparison between AJUBA‐high and AJUBA‐low groups further demonstrated significantly increased proportions of M0, M1, and M2 macrophages in AJUBA‐high tumors (Figure 2D). Together, these findings indicate that AJUBA may function as a regulator promoting macrophage infiltration or polarization within the colorectal tumor microenvironment.

Figure 2Evaluation of immune cell infiltration in relation to AJUBA expression levels. (A) Examination of the association between AJUBA expression and immune infiltration scores, as determined by single‐sample gene set enrichment analysis (ssGSEA). (B) Comparative analysis of immune cell infiltration levels, estimated through ssGSEA, among patients stratified according to either high or low AJUBA expression. (C) Scatterplot illustrating the correlation between AJUBA expression and the abundance of various immune‐infiltrating cell types, analyzed using the CIBERSORT algorithm. (D) Quantitative assessment of differences in the proportions of immune cell infiltration, calculated by CIBERSORT, between patient cohorts with high and low AJUBA expression.  ^∗^ *p* < 0.05;  ^∗∗^ *p* < 0.01;  ^∗∗∗^ *p* < 0.001; ns, not significant.

**Figure:**
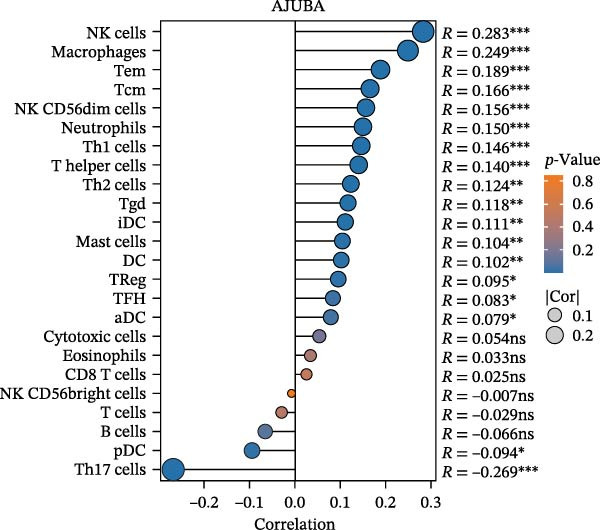
(A)

**Figure:**
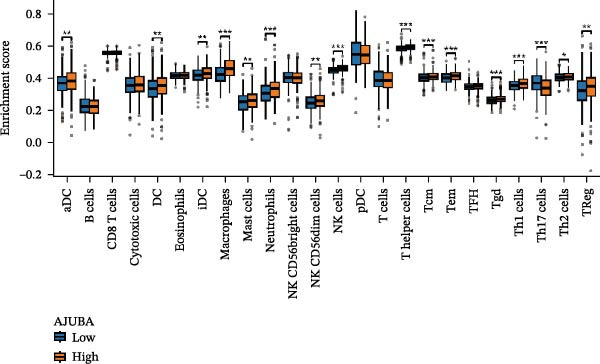
(B)

**Figure:**
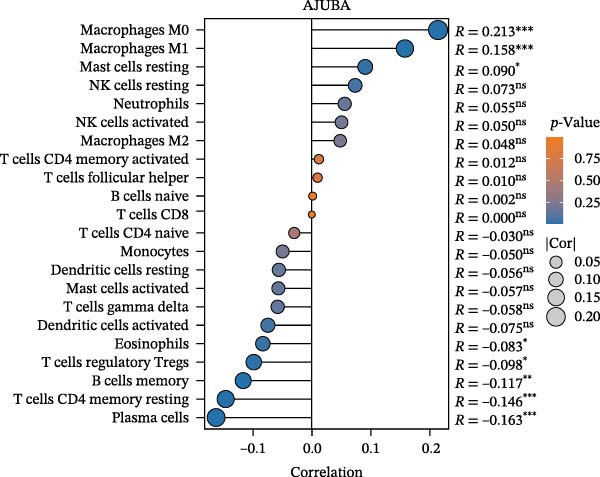
(C)

**Figure:**
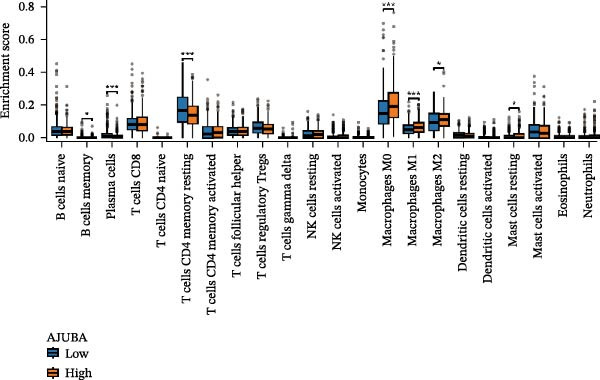
(D)

### 3.3. Analysis of AJUBA Expression Pattern at the Single‐Cell Level in CRC

Single‐cell RNA sequencing was performed using the GSE132465 CRC cohort after rigorous quality control, dimensionality reduction, and graph‐based clustering. Transcriptionally distinct clusters (*n* = 21) were delineated and categorized as 12 major cell types: dendritic cells, B cells, epithelial cells, cancer‐associated fibroblasts (CAFs), endothelial cells, glial cells, macrophages, mast cells, neutrophils, NK cells, plasma cells, and T cells (Figure 3A). Established marker genes employed for cell type annotation are provided in Figure 3C.

Figure 3Single‐cell analysis of AJUBA expression in colorectal cancer. (A) A UMAP (Uniform Manifold Approximation and Projection) plot that visualizes the cellular composition of colorectal cancer, revealing 12 clearly defined cell clusters. (B) A stacked bar chart that shows the relative abundance of various cell types in both tumor tissues and their corresponding normal tissues collected from colorectal cancer patients. (C) A dot plot presenting the key marker genes used to identify and annotate different cell types; the size of each dot indicates the proportion of cells within a cluster that express the respective gene, and the color intensity corresponds to the level of gene expression. (D) Feature plots that illustrate the spatial distribution and expression levels of AJUBA across diverse cell lineages, allowing for precise measurement of its transcript levels at the single‐cell level. (E, F) Comparative evaluation of AJUBA expression levels between colorectal cancer tissues and normal pancreatic tissues, depicted through a feature plot (E) and a violin plot (F).  ^∗^ *p* < 0.05; ns, not significant.

**Figure:**
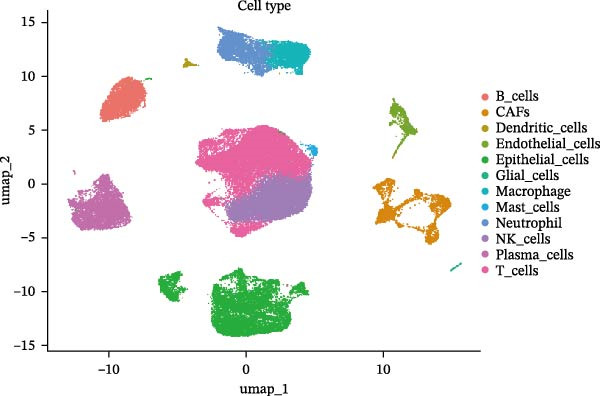
(A)

**Figure:**
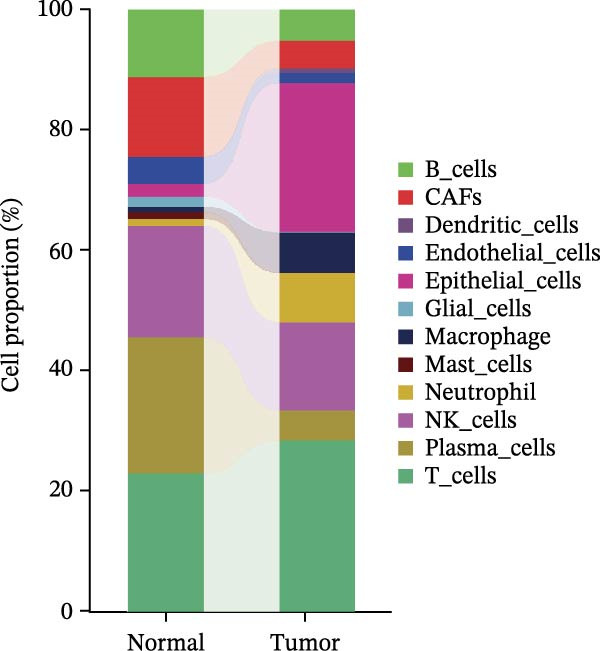
(B)

**Figure:**
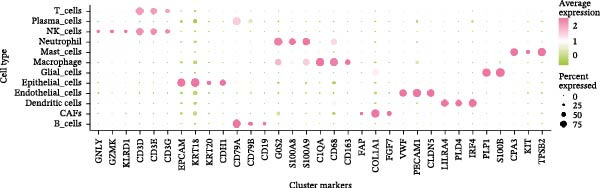
(C)

**Figure:**
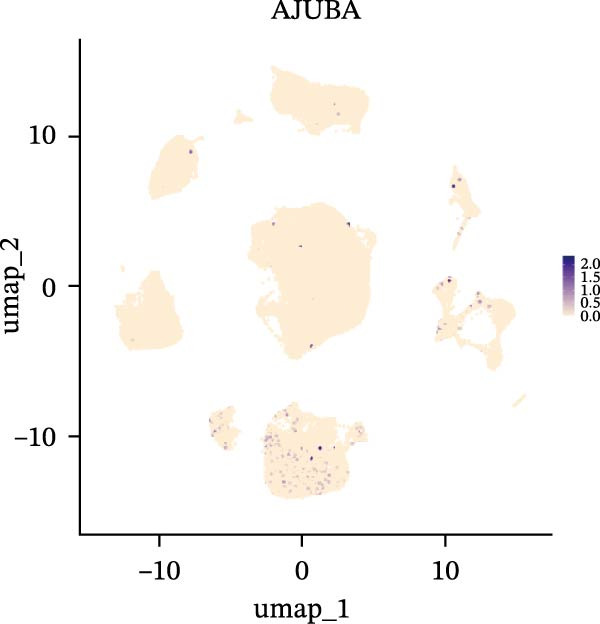
(D)

**Figure:**
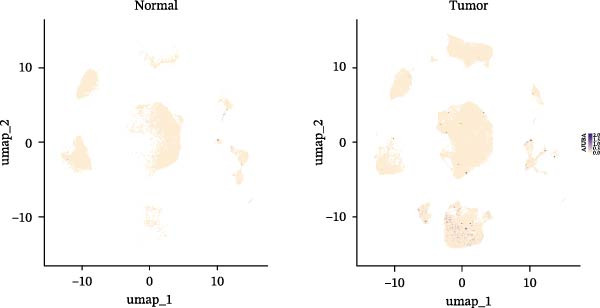
(E)

**Figure:**
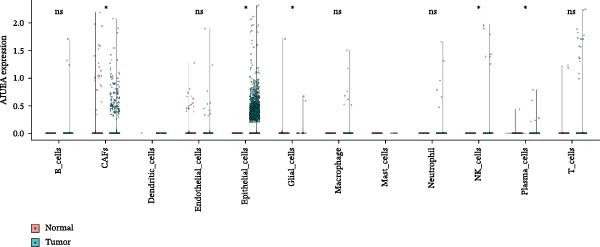
(F)

Analysis of cellular composition uncovered differential cellular distributions between tumor and adjacent normal tissues (Figure 3B). Normal tissue samples exhibited enrichment of plasma cells, NK cells, endothelial cells, and B cells, while tumor tissues displayed substantial elevations in T cells, neutrophils, macrophages, and epithelial cells, underscoring tumor microenvironment heterogeneity. AJUBA expression was detected in diverse cellular lineages, including CAFs, endothelial cells, epithelial cells, macrophages, neutrophils, NK cells, plasma cells, T cells, and B cells (Figure 3D). Additionally, comparative evaluations between matched tissue types revealed significantly elevated AJUBA transcript abundances in CAFs, epithelial cells, NK cells, and plasma cells relative to normal controls (Figure 3E, F).

### 3.4. Spatial Transcriptomic Analysis of AJUBA Expression

Four tumor samples—GSM7058756, GSM7058757, GSM7058758, and GSM7058759—were selected from the CRC spatial transcriptomics dataset GSE225857. Count data distributions for these specimens are displayed in Figure 4A–D. Following quality control procedures, spatial transcriptomic data were normalized and clustering parameters optimized to achieve precise spatial reconstruction. Spatial clustering delineated distinct regions within tissue sections: GSM7058756 yielded 10 clusters (Figure 4E), GSM7058757 produced 10 (Figure 4F), GSM7058758 generated 12 (Figure 4G), and GSM7058759 resulted in 9 (Figure 4H).

Figure 4Spatial transcriptomic analysis of AJUBA gene expression across distinct cell clusters in colorectal cancer. (A–D) Quantitative analysis of spatially resolved transcriptomic spots in the tissue specimens identified as GSM7058756, GSM7058757, GSM7058758, and GSM7058759. (E–H) Identification and classification of spatial transcriptomic clusters within the corresponding samples GSM7058756, GSM7058757, GSM7058758, and GSM7058759. (I–L) Visualization and quantification of AJUBA mRNA distribution patterns specifically within each identified cluster in samples GSM7058756, GSM7058757, GSM7058758, and GSM7058759. (M–P) Inferred cellular composition of each sample based on deconvolution analysis, highlighting the proportional representation of different cell types in specimens GSM7058756, GSM7058757, GSM7058758, and GSM7058759. (Q–T) Spatial mapping of AJUBA expression levels and evaluation of its expression heterogeneity across the analyzed tissue samples GSM7058756, GSM7058757, GSM7058758, and GSM7058759.

**Figure:**
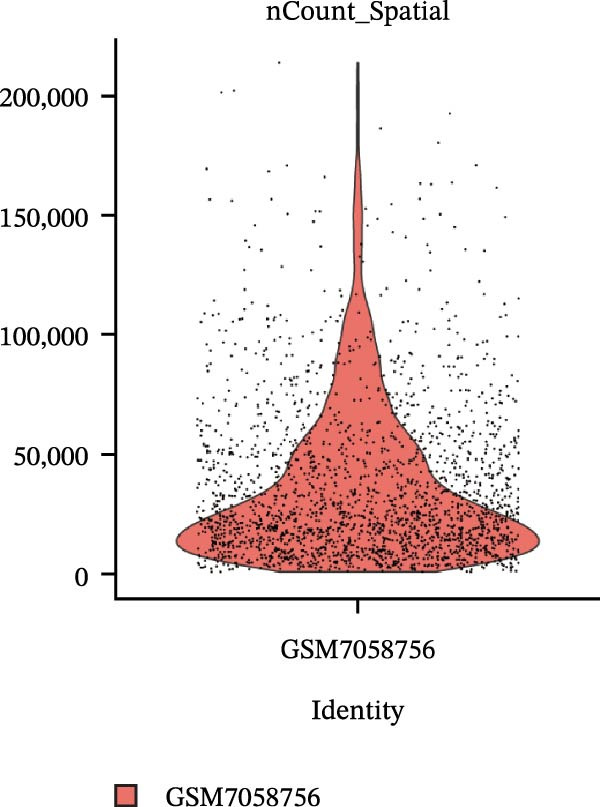
(A)

**Figure:**
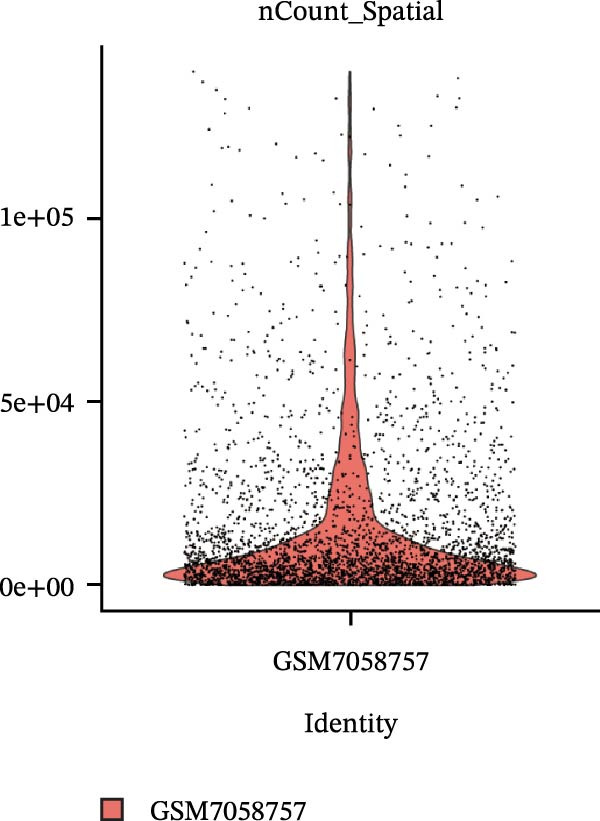
(B)

**Figure:**
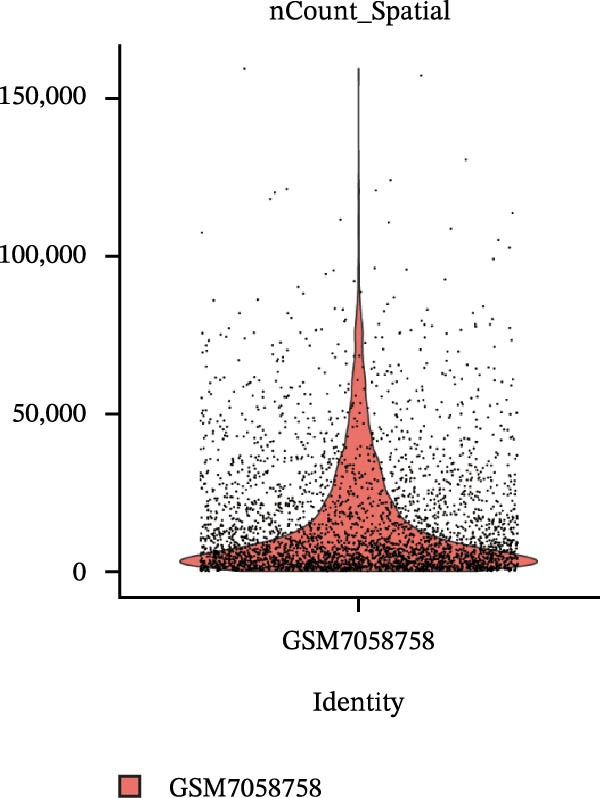
(C)

**Figure:**
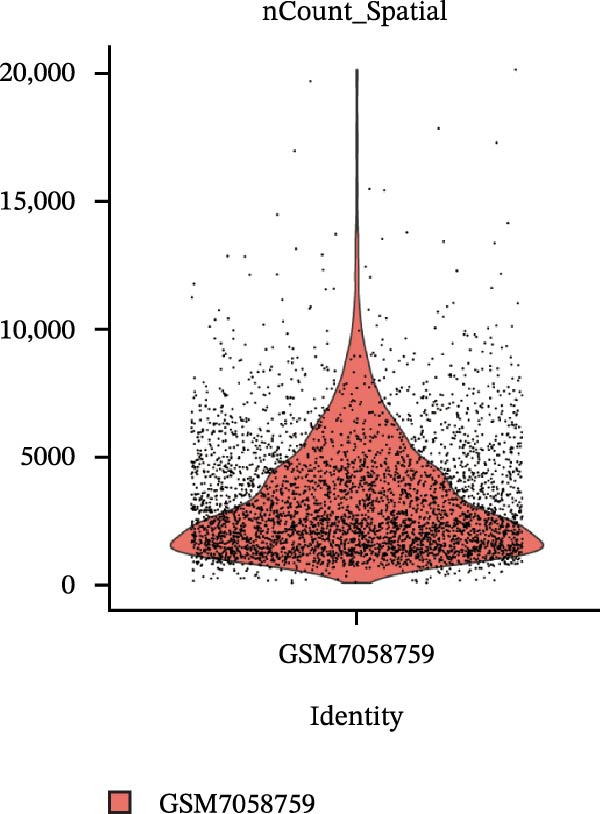
(D)

**Figure:**
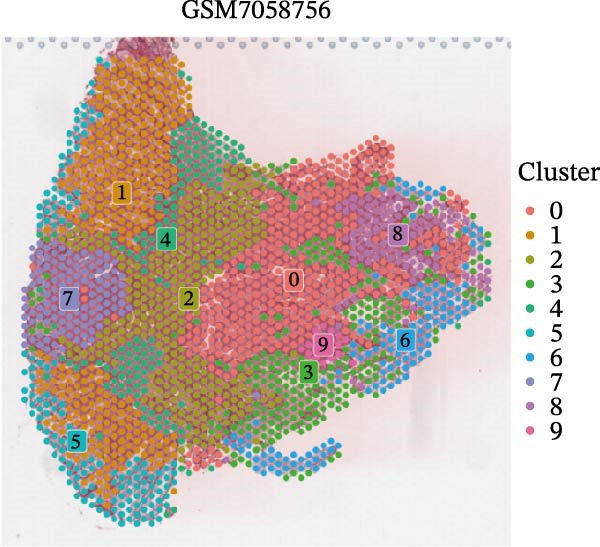
(E)

**Figure:**
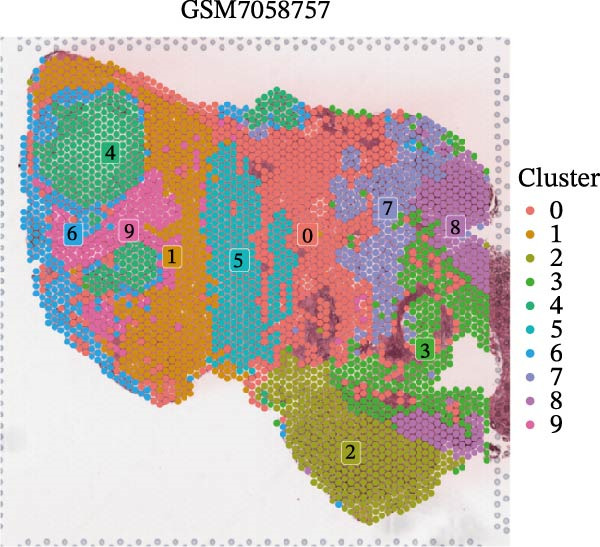
(F)

**Figure:**
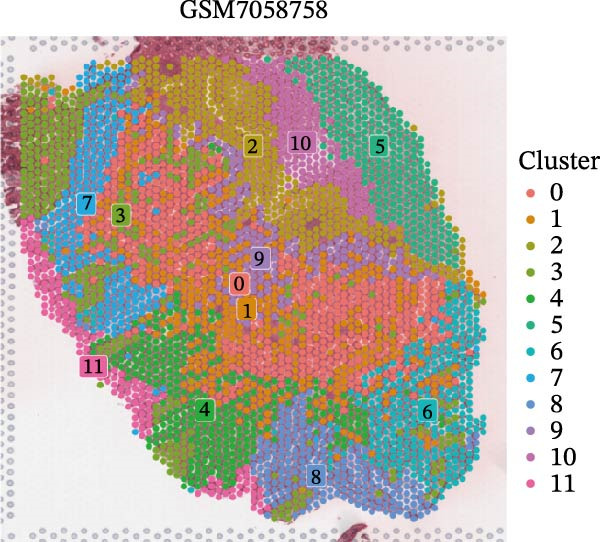
(G)

**Figure:**
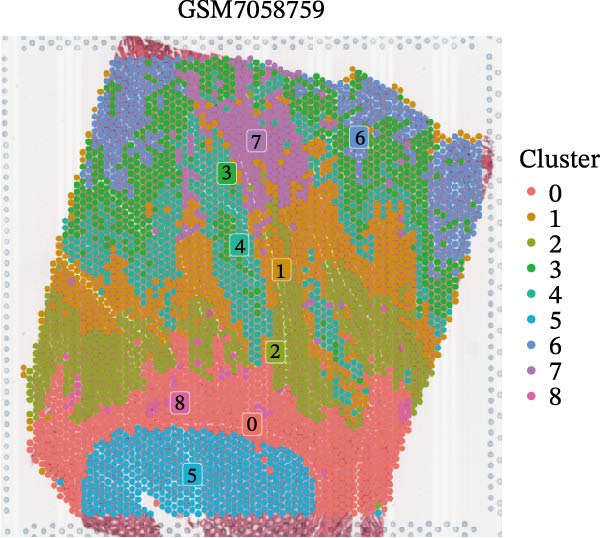
(H)

**Figure:**
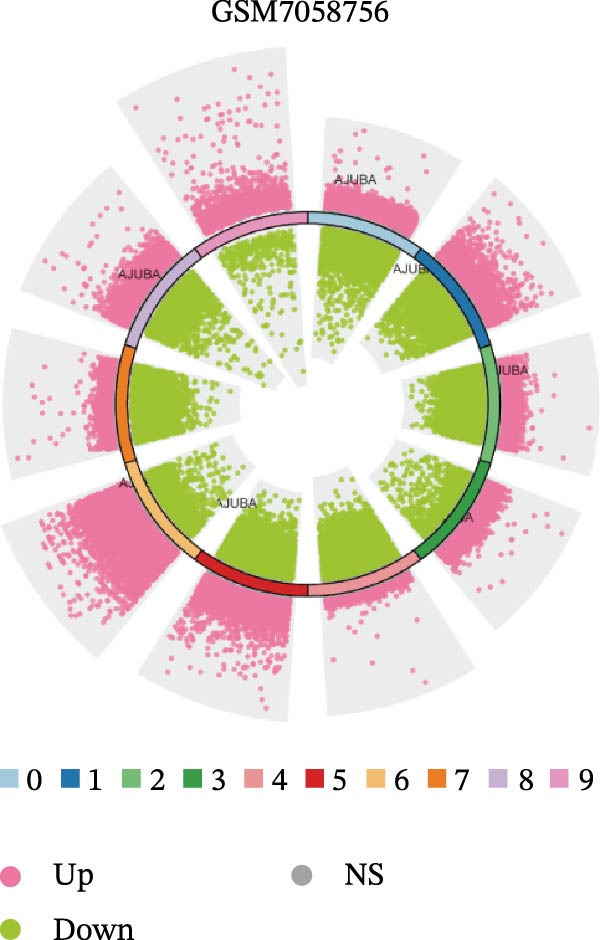
(I)

**Figure:**
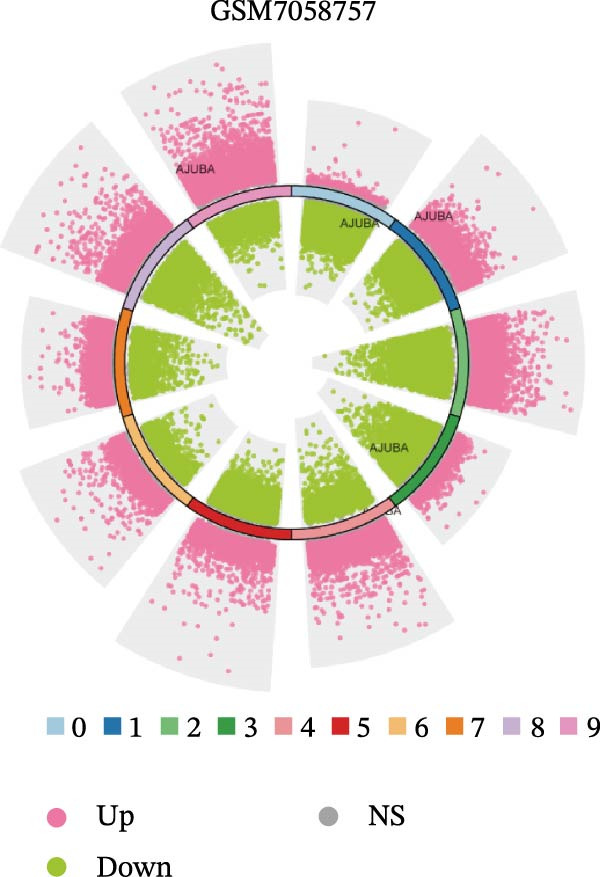
(J)

**Figure:**
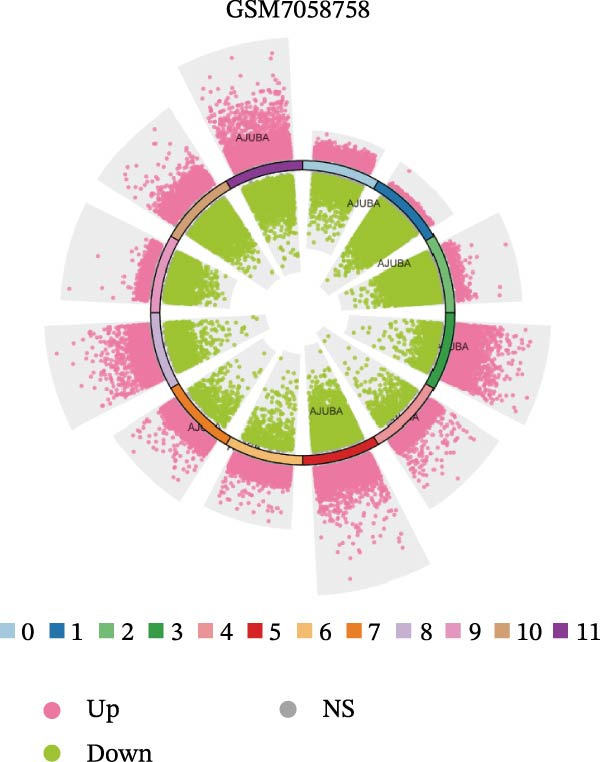
(K)

**Figure:**
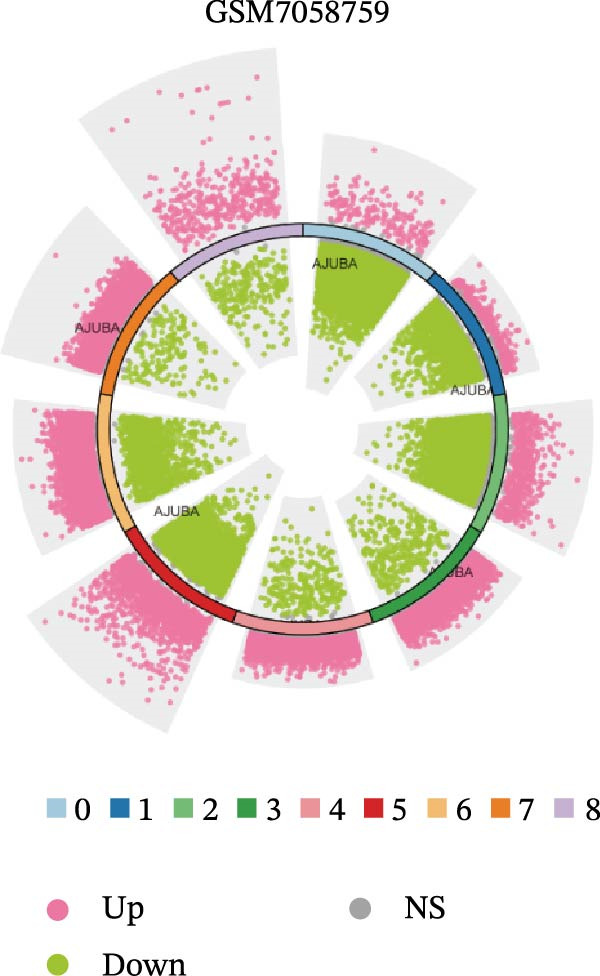
(L)

**Figure:**
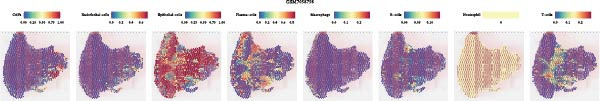
(M)

**Figure:**
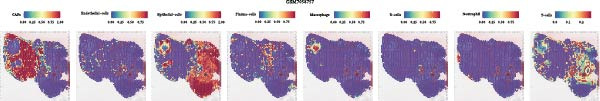
(N)

**Figure:**
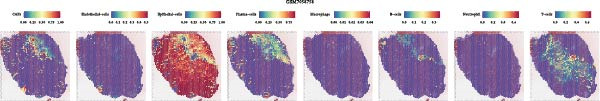
(O)

**Figure:**
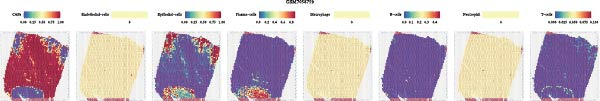
(P)

**Figure:**
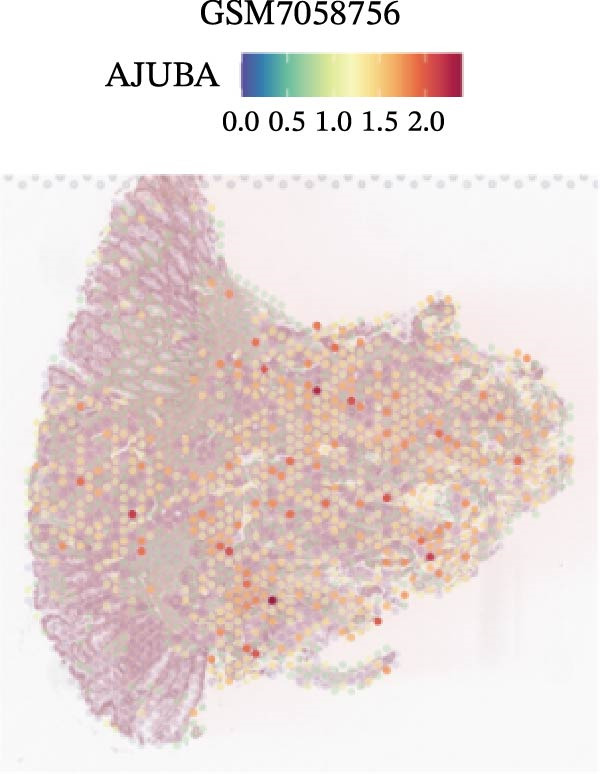
(Q)

**Figure:**
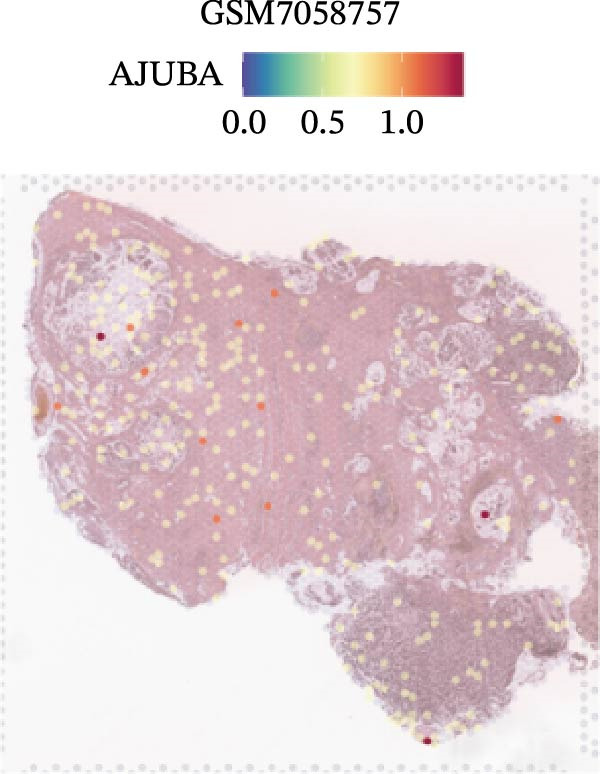
(R)

**Figure:**
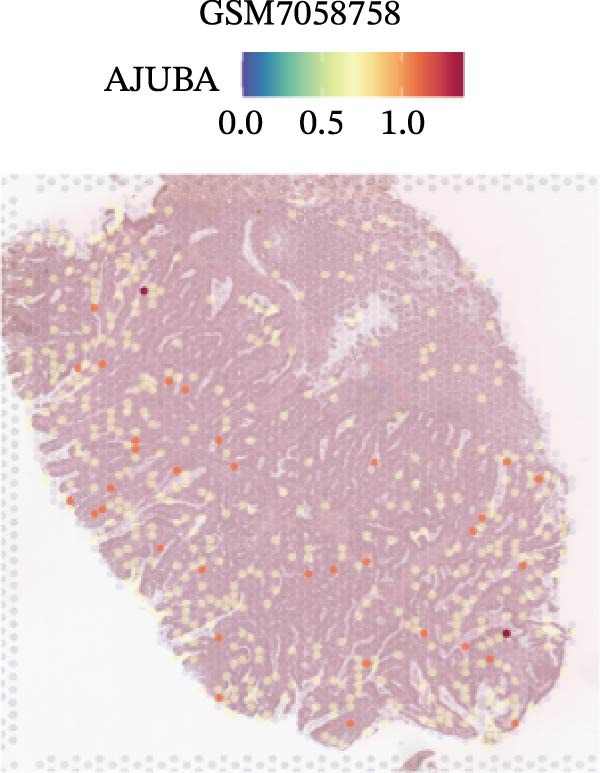
(S)

**Figure:**
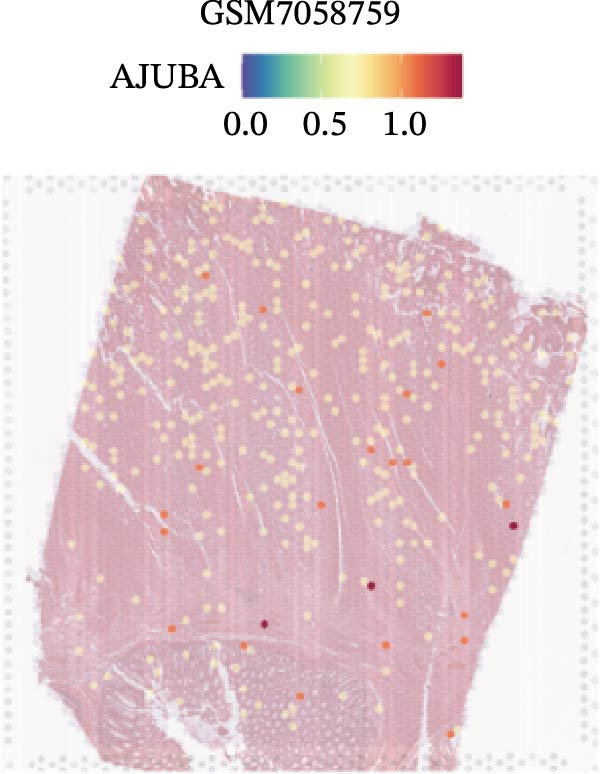
(T)

To identify spatially variable genes, differentially expressed genes were determined across spatial clusters in each sample. The AJUBA expression was elevated in specific clusters: clusters 0, 2, 3, 6, and 8 in GSM7058756 (Figure 4I); clusters 1, 4, and 9 in GSM7058757 (Figure 4J); clusters 3, 4, 6, 7, and 11 in GSM7058758 (Figure 4K); and clusters 3, 4, and 7 in GSM7058759 (Figure 4L). For estimating cellular composition within spatial domains, cellular deconvolution was performed using a reference single‐cell RNA sequencing dataset. Spatially resolved cell type assignments for the four samples appear in Figure 4M–P. Spatial mapping of AJUBA expression revealed primary localization to epithelial cells and CAFs across all specimens (Figure 4Q–T), consistent with prior single‐cell transcriptomic observations.

### 3.5. Functional Consequences of AJUBA Expression in Neoplastic Epithelial Cells

Cells were categorized into AJUBA‐high and AJUBA‐low subsets according to median AJUBA expression levels. The AJUBA‐high subset displayed a significantly elevated representation of epithelial cells and CAFs (Figure 5A). Epithelial cells were further classified as AJUBA+ (AJUBA‐expressing) or AJUBA− (AJUBA‐nonexpressing) and relabeled (Figure 5B). Comparative analysis of gene expression between AJUBA + and AJUBA− epithelial cells was conducted, followed by application of Gene Set Enrichment Analysis (GSEA) to the differentially expressed genes. This approach identified heightened activity of multiple pivotal pathways in AJUBA + epithelial cells, including VEGFA/VEGFR2 signaling, programed cell death, MAPK cascades, PI3K‐AKT signaling, NOTCH signaling, and EMT in CRC (Figure 5C). Relevant gene sets for these six pathways were acquired from the GSEA database (MSigDB) to perform enrichment analysis at single‐cell resolution. UMAP visualizations in Figures 5D–I illustrate enrichment scores for each pathway. The data reveal pronounced upregulation of all six pathways in AJUBA + epithelial cells relative to AJUBA− cells (Figure 5J).

Figure 5Functional roles of AJUBA expression in cancerous epithelial cells. (A) A circular bar chart displaying the relative distribution of cell populations based on AJUBA expression levels, stratified into high (equal to or above the median) and low (below the median) categories across all analyzed samples. (B) A UMAP (Uniform Manifold Approximation and Projection) plot visualizing epithelial cells, which were further re‐clustered and distinguished into AJUBA‐positive and AJUBA‐negative subsets for clearer visualization. (C) Results from gene set enrichment analysis (GSEA) comparing the gene expression signatures of epithelial cell populations with high AJUBA expression versus those with low AJUBA expression. (D–I) Spatial mapping of pathway enrichment scores displayed within the context of the UMAP projections, highlighting the distribution of activated signaling pathways across different cellular clusters. (J) A dot plot illustrating both the activation status and the percentage of cells expressing gene signatures associated with the six most significantly upregulated Hallmark signaling pathways.

**Figure:**
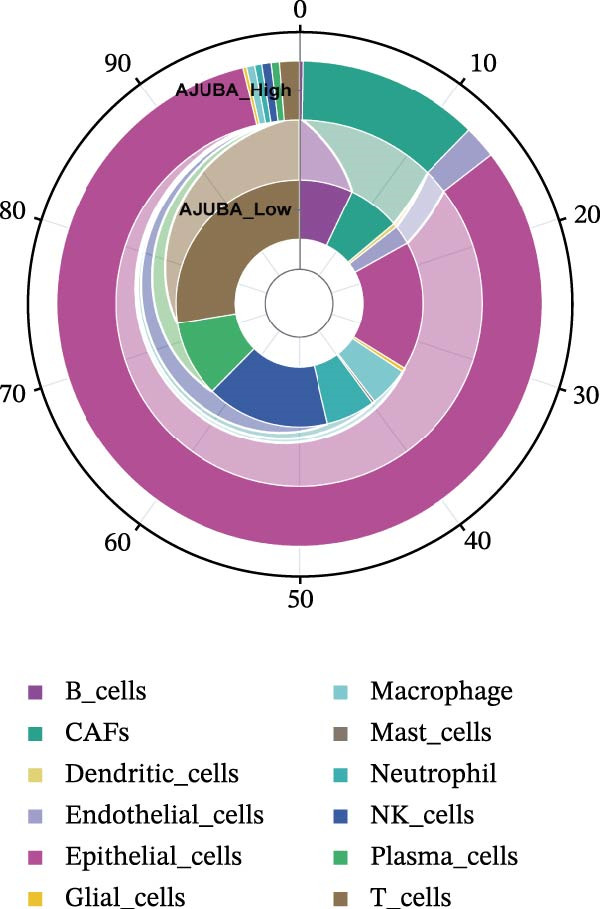
(A)

**Figure:**
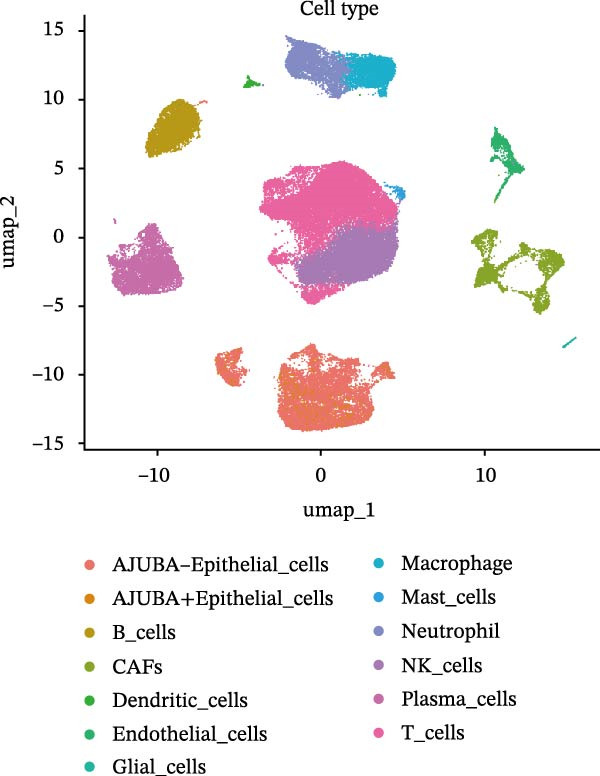
(B)

**Figure:**
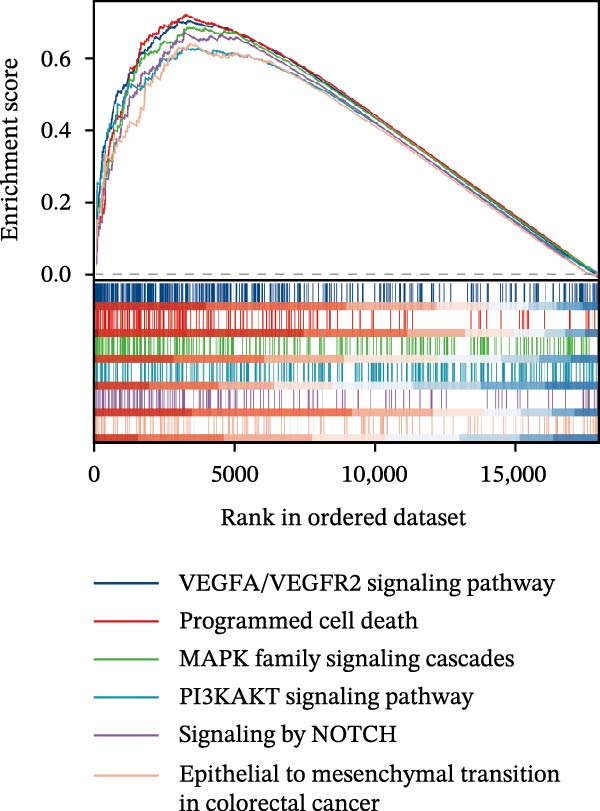
(C)

**Figure:**
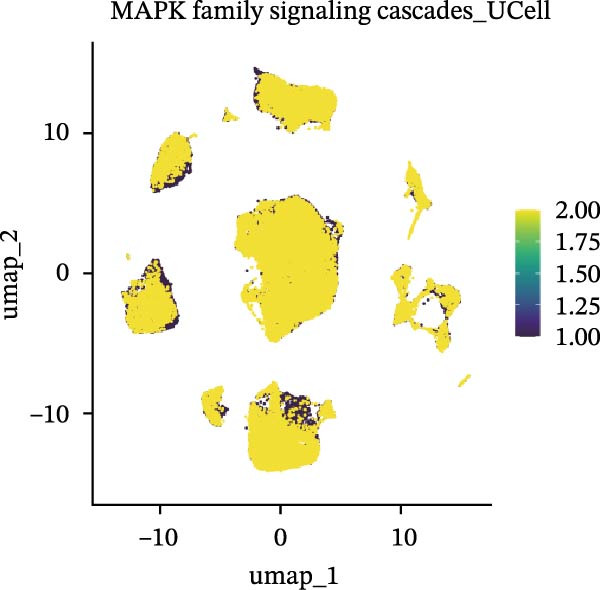
(D)

**Figure:**
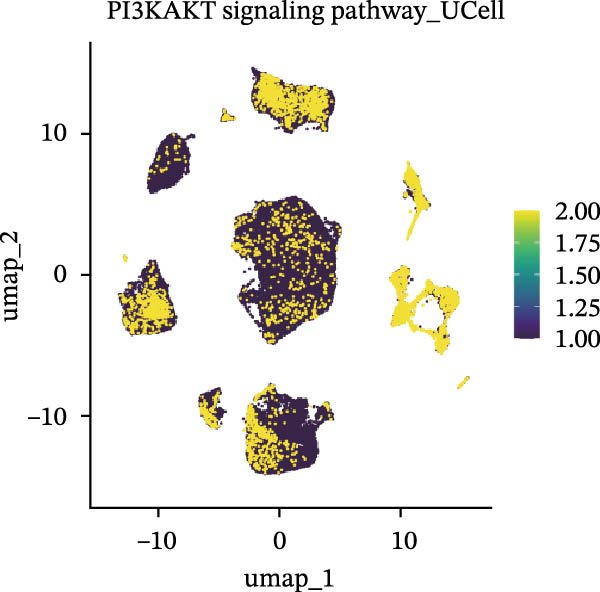
(E)

**Figure:**
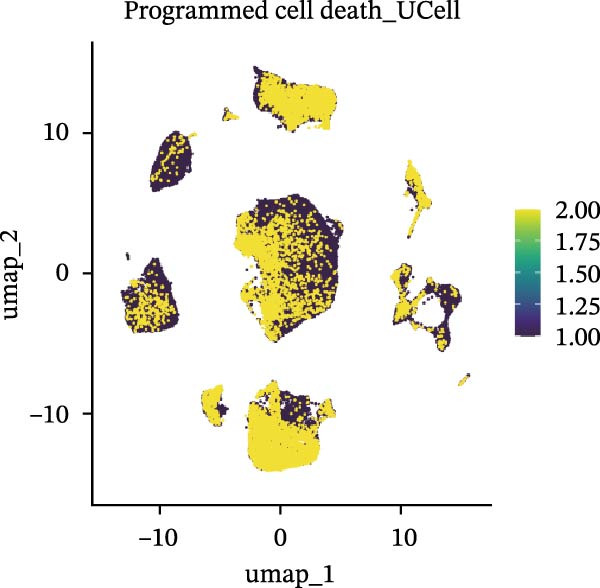
(F)

**Figure:**
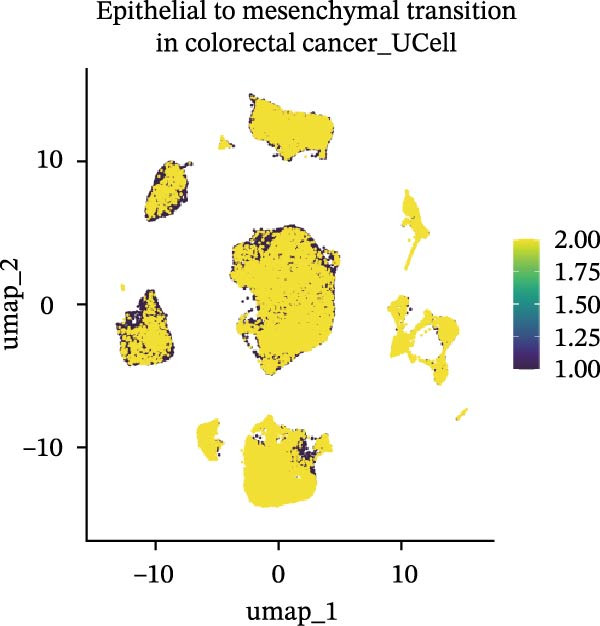
(G)

**Figure:**
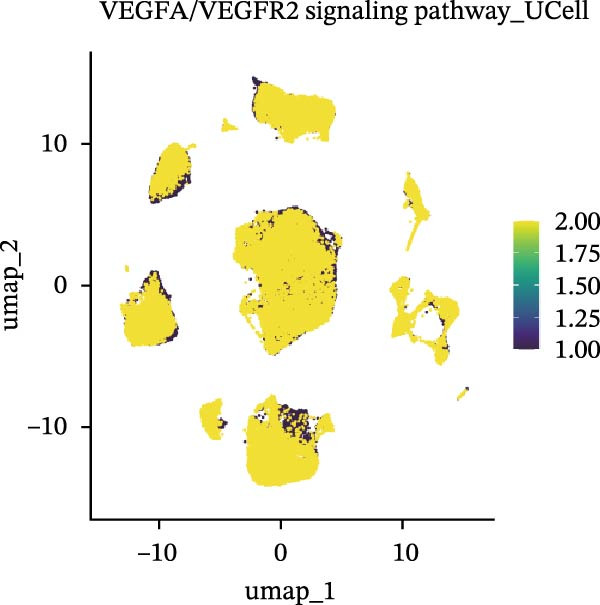
(H)

**Figure:**
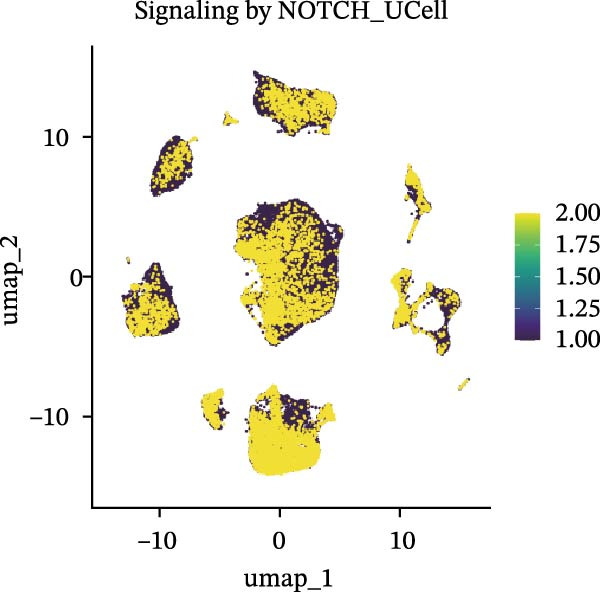
(I)

**Figure:**
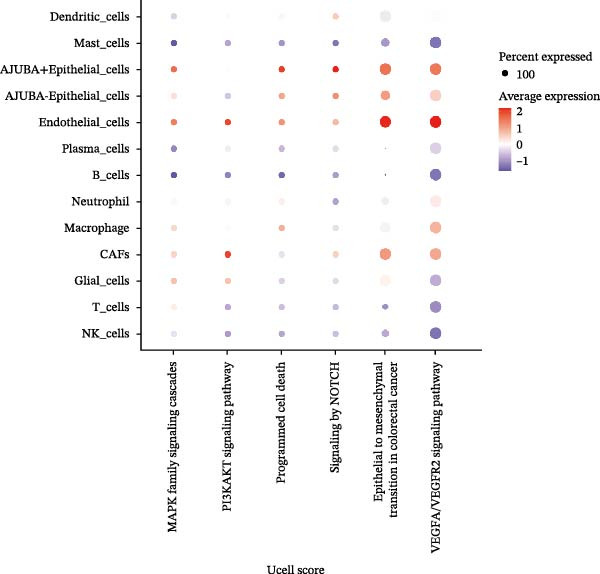
(J)

### 3.6. Differential Intercellular Communication Networks of AJUBA+ Versus AJUBA− Epithelial Cells in CRC

To elucidate AJUBA’s functional contributions to cell–cell signaling, we applied CellChat to examine communication networks between AJUBA‐positive and AJUBA‐negative cellular subsets. The evaluation indicated substantially diminished interaction numbers and lower signaling intensity in normal tissues compared to tumor samples. Within tumor microenvironments, AJUBA‐expressing epithelial cells displayed heightened interaction rates and stronger communication capacity relative to AJUBA‐nonexpressing epithelial cells (Figure 6A–D).

Figure 6The regulatory role of AJUBA in the colorectal cancer microenvironment. (A, B) A radial plot illustrating the frequency of intercellular communication events among different cell types in normal (A) and tumor (B) tissues. (C, D) Circular diagrams representing the strength of signaling interactions between cellular clusters in normal (C) and cancerous (D) tissue samples. (E–J) Heatmap visualizations displaying the likelihood of interactions between distinct cellular subpopulations across various signaling pathways. (E, F) Heatmaps specifically depicting the interaction networks of the MIF signaling pathway in normal (E) and malignant (F) tissues. (G, H) Heatmaps showing the communication probabilities associated with the NOTCH signaling pathway in normal (G) and tumor (H) environments. (I, J) Heatmaps illustrating the potential for interactions involving AJUBA‐positive and AJUBA‐negative epithelial cells with other cell types, shown for normal (I) and cancerous (J) tissue contexts.

**Figure:**
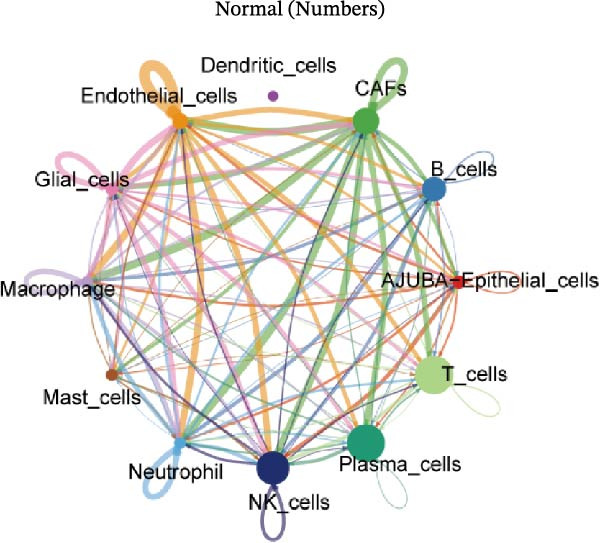
(A)

**Figure:**
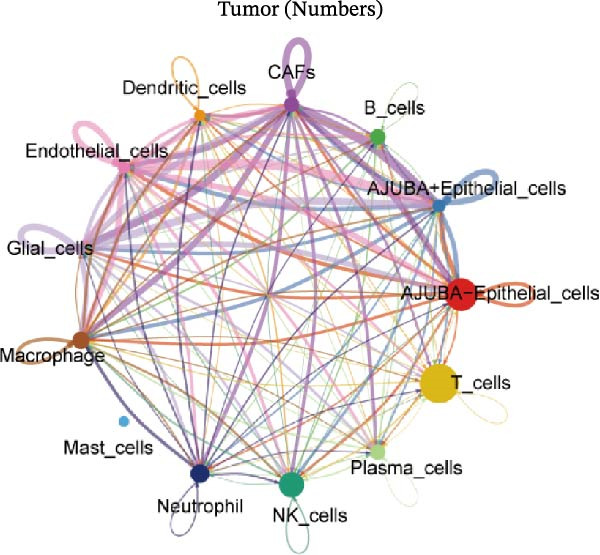
(B)

**Figure:**
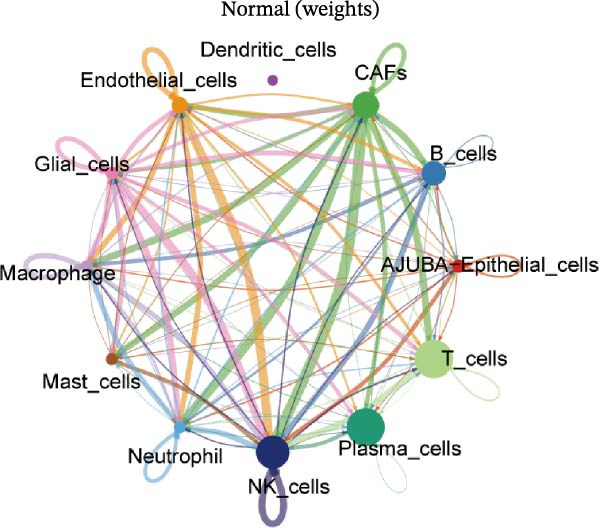
(C)

**Figure:**
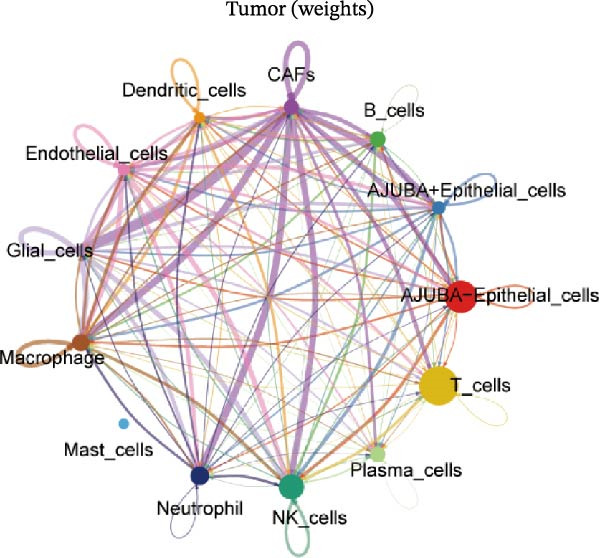
(D)

**Figure:**
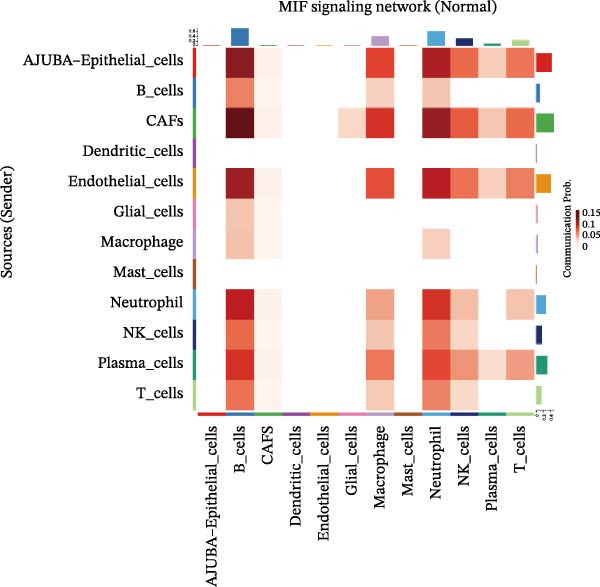
(E)

**Figure:**
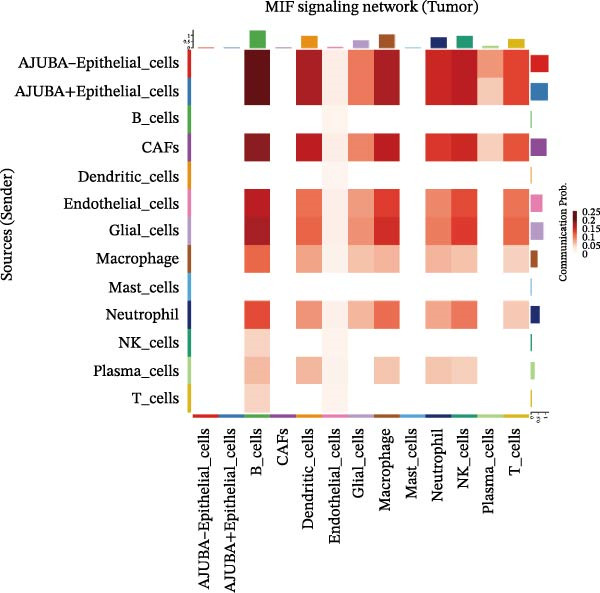
(F)

**Figure:**
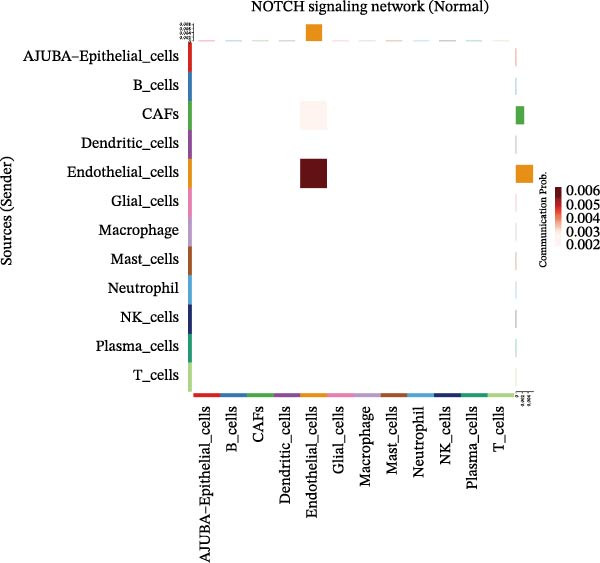
(G)

**Figure:**
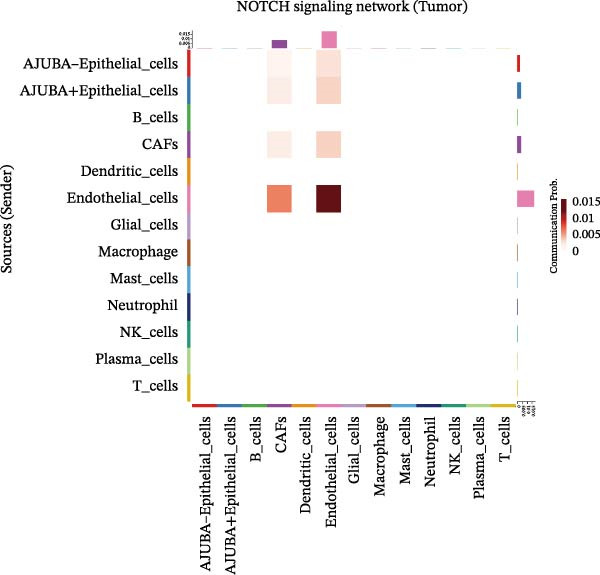
(H)

**Figure:**
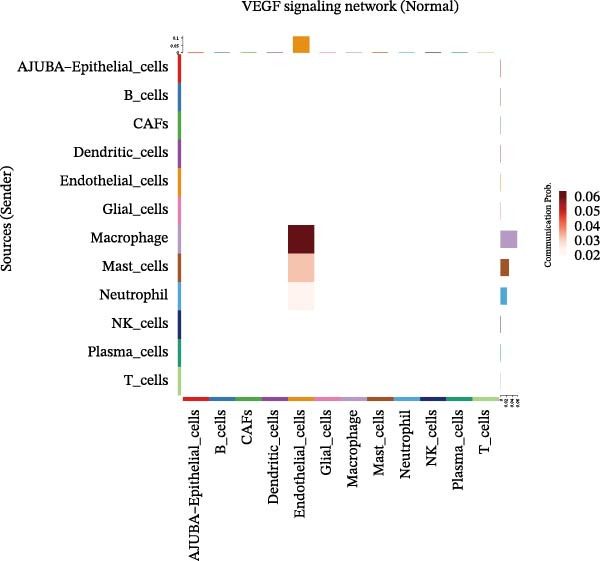
(I)

**Figure:**
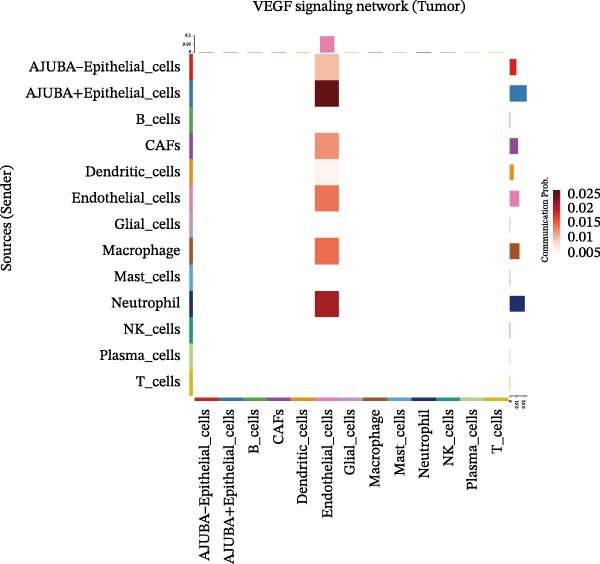
(J)

Based on earlier GSEA results, which indicated heightened activity in the NOTCH and VEGF signaling pathways, we chose to focus on these two pathways along with the MIF pathway—an important molecular cascade that has been closely associated with cancer development—for visual representation through heatmap analysis. In normal tissue samples, epithelial cells showed increased interaction probabilities with various types of immune cells, including B lymphocytes, macrophages, neutrophils, natural killer (NK) cells, and T lymphocytes (Figure 6E,F). In tumor samples, regardless of whether the epithelial cells expressed AJUBA or not, they continued to display similarly elevated chances of communicating with these same immune cell populations. Moreover, there were only slight differences in communication likelihoods between the AJUBA‐positive and AJUBA‐negative groups (Figure 6E,F).

In healthy tissues, epithelial cells lacking AJUBA expression exhibited minimal involvement in signaling interactions associated with both the NOTCH and VEGF pathways (Figure 6G–J). In contrast, within tumor tissues, epithelial cells that were positive for AJUBA demonstrated a significantly greater likelihood of engaging in signaling interactions compared to their AJUBA‐negative counterparts, particularly within the VEGF signaling cascade, where the difference was most striking (Figure 6G–J). As a result, the VEGF pathway was selected for more detailed investigation.

The examination of the VEGF signaling network demonstrated an absence of observable interactions between AJUBA‐negative epithelial cells and endothelial cells in healthy tissues (Figure 7A,B). Conversely, within tumor samples, epithelial cells, regardless of their AJUBA expression status, engaged in interactions with endothelial cells, with AJUBA‐positive variants exhibiting significantly heightened interaction intensities (Figure 7A,B). An analysis of specific ligand–receptor combinations, such as VEGFA–VEGFR1 and VEGFA–VEGFR2, corroborated these findings: no interactions were found in normal tissues, while both ligand–receptor pairs were active within tumor microenvironments, with AJUBA‐positive epithelial cells revealing increased interaction strength (Figure 7C–H).

Figure 7Modulation of VEGF signaling pathways by epithelial cells expressing or lacking AJUBA in both normal and tumor microenvironments. (A,B) An integrated signaling network involving VEGF, depicting the interactive dynamics between AJUBA‐positive and AJUBA‐negative epithelial cells along with all other cell types present in the normal (A) and tumor (B) microenvironments. (C–E) Ligand–receptor interaction profiles observed in normal tissue: (C) interactions between VEGFA and VEGFR1, (D) communication events mediated by VEGFA with both VEGFR1 and VEGFR2, (E) direct interactions between VEGFA and VEGFR2. (F–H) Ligand–receptor interaction patterns that are more prominent in tumor tissue: (F) associations between VEGFA and VEGFR1, (G) signaling events involving VEGFA and the combined VEGFR1 and VEGFR2 receptors, (H) interaction characteristics specific to VEGFA and VEGFR2.

**Figure:**
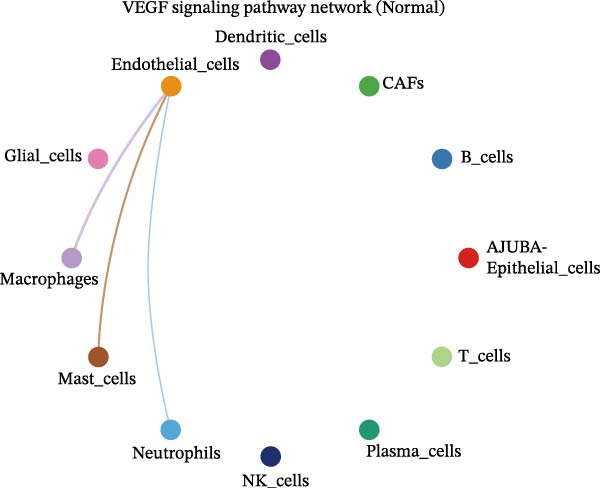
(A)

**Figure:**
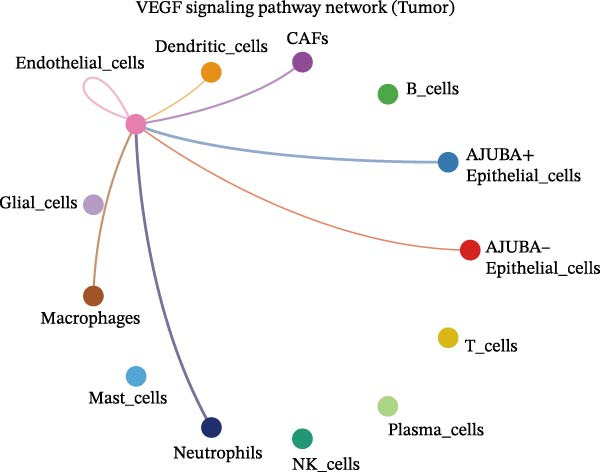
(B)

**Figure:**
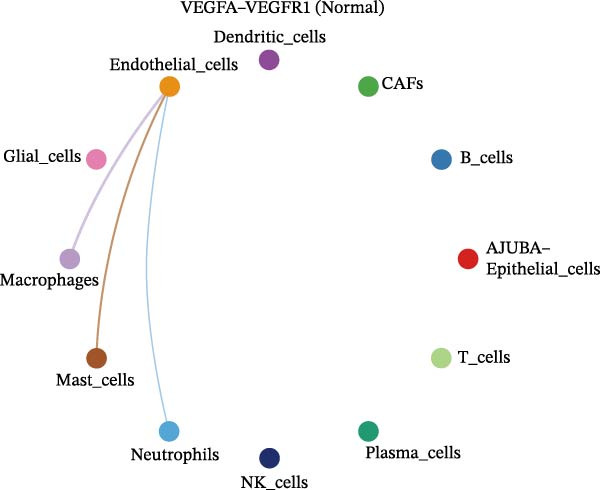
(C)

**Figure:**
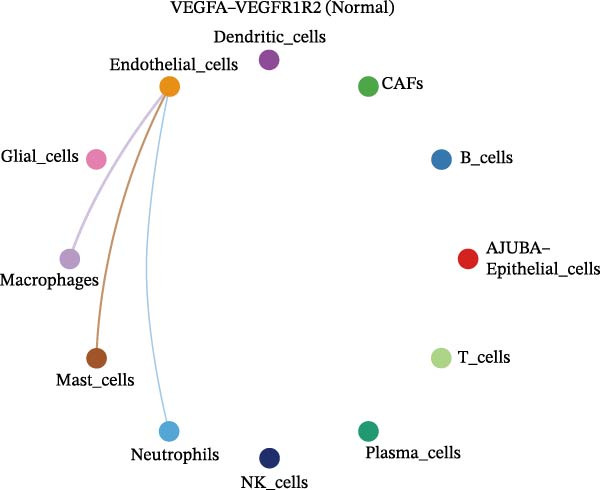
(D)

**Figure:**
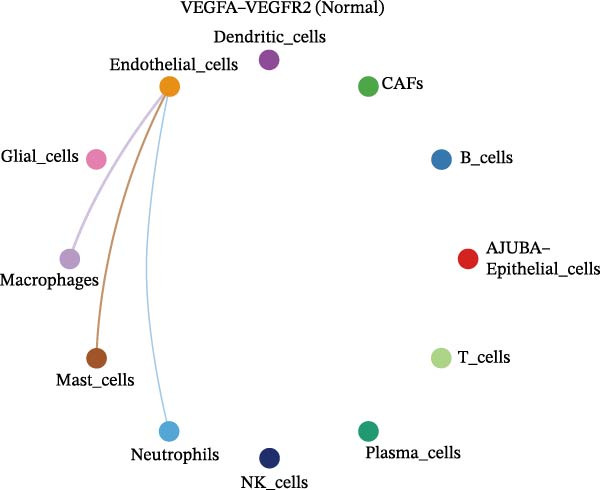
(E)

**Figure:**
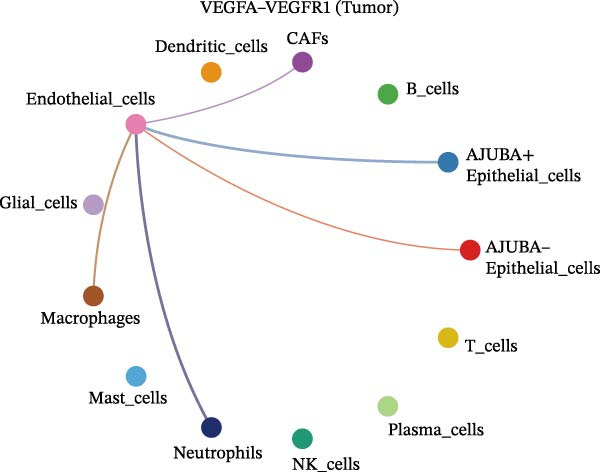
(F)

**Figure:**
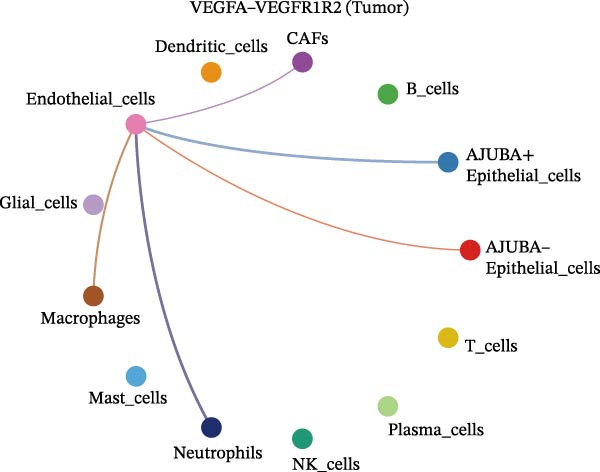
(G)

**Figure:**
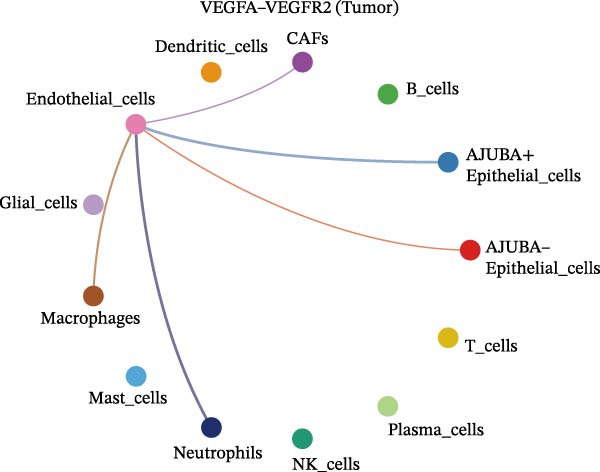
(H)

Subsequent quantitative analyses were performed to assess directional signaling dynamics across distinct cellular subsets. Ligand–receptor‐mediated communication exhibited maximal inward signal transduction within NK cell populations [1]. Notably, epithelial subsets positive for AJUBA demonstrated significantly elevated inward signaling flux relative to their AJUBA‐negative counterparts (Figure 8A). Comprehensive heatmapping of inferred signaling networks further delineated functional asymmetries, wherein AJUBA‐positive epithelial cells functioned as both predominant signal emitters and recipients, contrasting with the reduced signaling activity observed in AJUBA‐negative populations (Figure 8B).

Figure 8Distinct VEGF‐mediated intercellular communication networks orchestrated by AJUBA‐expressing versus AJUBA‐deficient epithelial cells within the tumor microenvironment. (A) A comparative analysis of the intensity of VEGF signal reception and emission across key cell populations, with a focus on highlighting the differences observed between epithelial cells that express AJUBA and those that lack AJUBA expression in the tumor setting. (B) A heatmap displaying quantitative measures of network centrality, which reflect the relative importance of each cell type in the propagation and reception of VEGF signals within the cellular network. (C) Schematic visualizations of VEGF signaling pathways, depicting specific ligand–receptor interaction events that occur between AJUBA‐positive and AJUBA‐negative epithelial cell populations. (D) Heatmaps organized by signaling pathway, illustrating the involvement of distinct cellular subsets in VEGF‐driven intercellular communication processes. (E) Output patterns of VEGF signaling emanating from AJUBA^+^/^−^ epithelial cells directed at potential target cells within the tumor setting.

**Figure:**
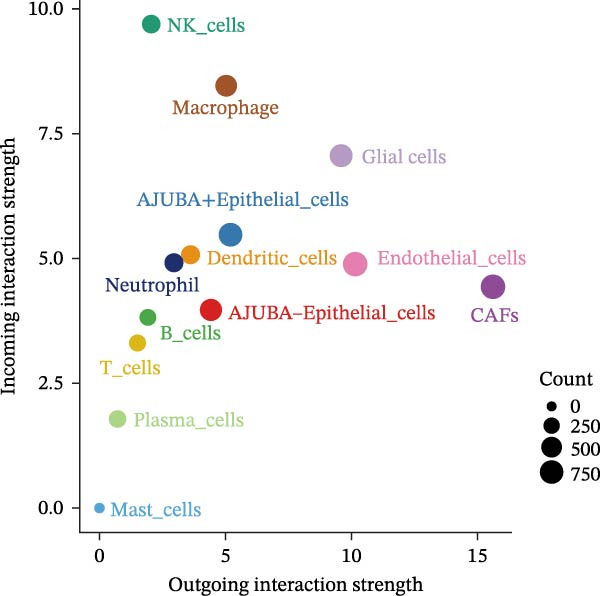
(A)

**Figure:**
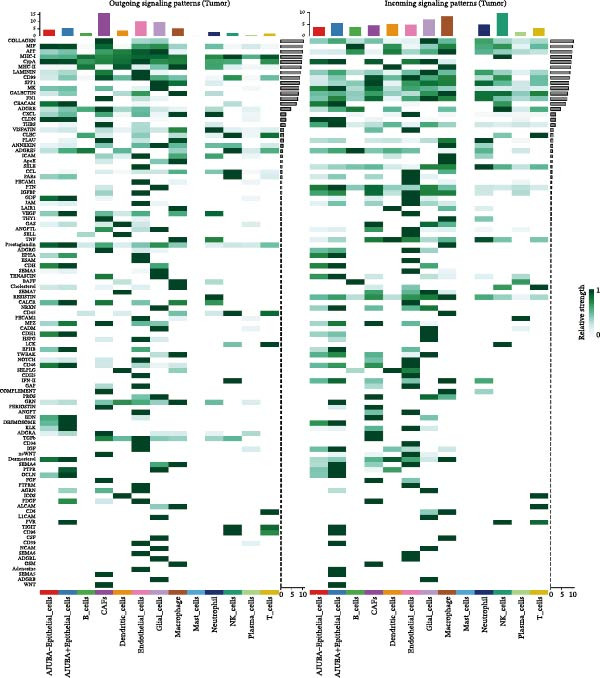
(B)

**Figure:**
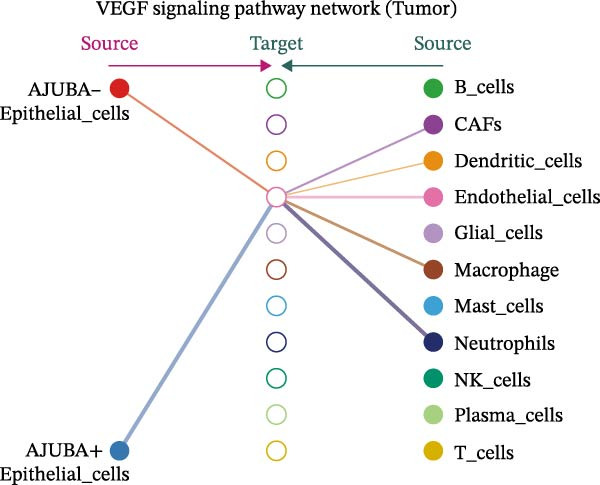
(C)

**Figure:**
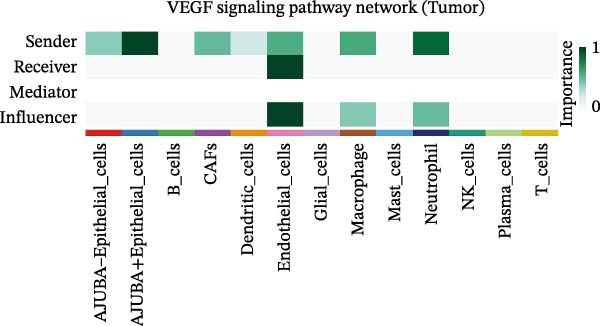
(D)

**Figure:**
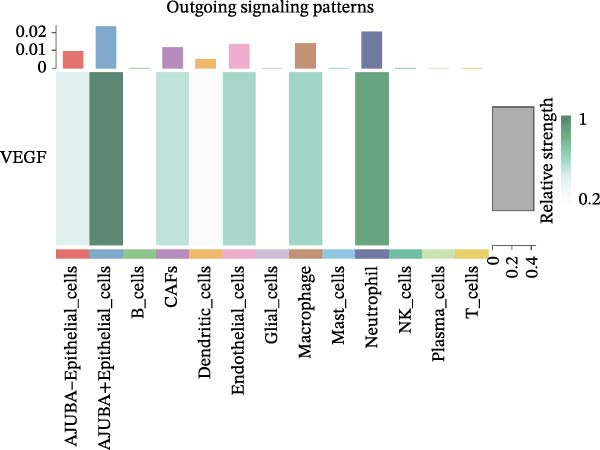
(E)

These observations substantiate the hypothesis that vascular endothelial growth factor (VEGF)‐mediated signaling plays an integral role in orchestrating tumor microenvironmental regulation. Network‐based interrogation of VEGF‐associated pathways confirmed that epithelial compartments, irrespective of AJUBA expression status, constituted primary signaling hubs engaged in bidirectional crosstalk with endothelial lineages—wherein AJUBA‐positive subsets exhibited comparatively heightened signaling engagement (Figure 8C). Intensive pathway deconvolution revealed that both AJUBA‐positive and AJUBA‐negative epithelial entities operated as functionally significant signal‐originating nodes within the tumoral milieu, displaying augmented expression signatures and mechanistic prominence, albeit with quantitatively superior metrics in AJUBA‐positive populations (Figure 8D). Parallel evaluations of efflux signaling dynamics identified consistently elevated outward signaling potency in AJUBA‐positive epithelial cells when compared to AJUBA‐negative counterparts (Figure 8E).

### 3.7. AJUBA is Highly Expressed in CRC

As illustrated in Figure 9A, quantitative assessment revealed a consistent upregulation of AJUBA transcript levels in all 10 colorectal tumor samples when compared with their corresponding noncancerous adjacent tissues. The degree of elevation ranged broadly, with fold‐change values spanning from ~1.6 to –33.9. Correspondingly, Figure 9B highlights a notable increase in AJUBA protein abundance within freshly acquired colorectal carcinoma specimens, in clear distinction to the protein expression detected in adjacent healthy mucosa. Further IHC characterization conducted on a cohort of 90 individuals diagnosed with colorectal adenocarcinoma demonstrated that AJUBA was overexpressed in a substantial proportion of cases, specifically in 55.56% (50 out of 90) of the evaluated tumor tissues. By contrast, the surrounding nonneoplastic tissues exhibited either minimal or undetectable AJUBA immunoreactivity, with only 18.89% (17 of 90) of these nonmalignant samples displaying positive staining signals. Statistical evaluation confirmed a highly significant divergence in AJUBA expression patterns between the neoplastic and adjacent nontumorous tissues (*χ* ^2^ = 24.202; *p* < 0.001).

Figure 9Comparative analysis of AJUBA mRNA and protein expression in colorectal cancer versus matched adjacent nontumor tissues. (A) Quantitative real‐time PCR was performed to measure AJUBA mRNA levels in 10 paired samples of colorectal cancer and adjacent normal tissues. The results demonstrated a statistically significant increase in AJUBA transcript levels in tumor tissues compared to their normal counterparts (*p* = 0.0017). In this figure, “Normal” refers to the adjacent noncancerous tissues, while “Tumor” represents colorectal cancer tissues. (B) Western blot analysis was conducted on the same 10 paired samples to evaluate AJUBA protein expression. The samples are labeled as follows: T for tumor tissue and ANT for adjacent nontumorous tissue. (C) Immunohistochemical staining showing no detectable AJUBA protein expression in normal colon mucosa. (D) Immunohistochemical images illustrating AJUBA expression in normal colon tissues. (E) Immunohistochemical staining indicating the absence of AJUBA immunoreactivity in colorectal cancer samples. (F) Immunohistochemical detection of AJUBA protein in colorectal carcinoma tissues.

**Figure:**
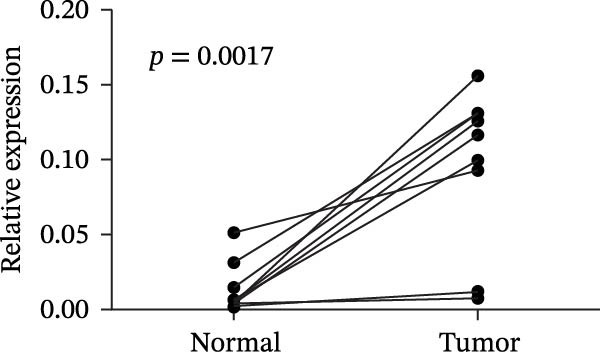
(A)

**Figure:**
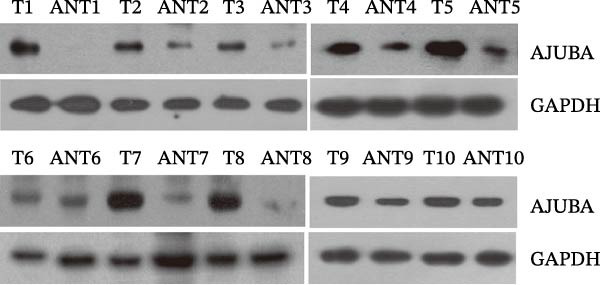
(B)

**Figure:**
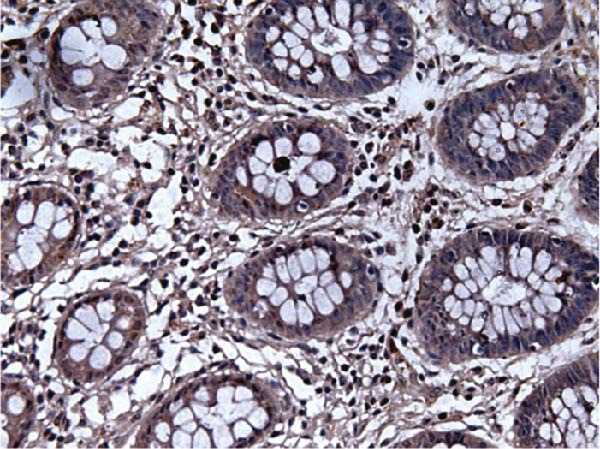
(C)

**Figure:**
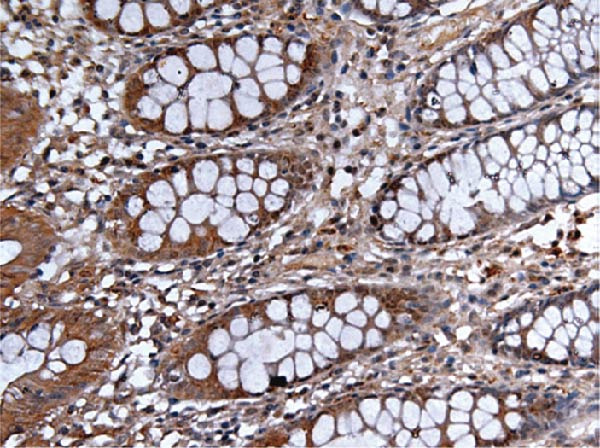
(D)

**Figure:**
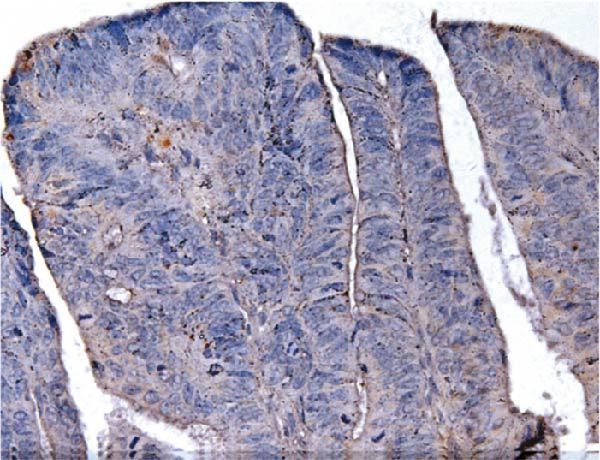
(E)

**Figure:**
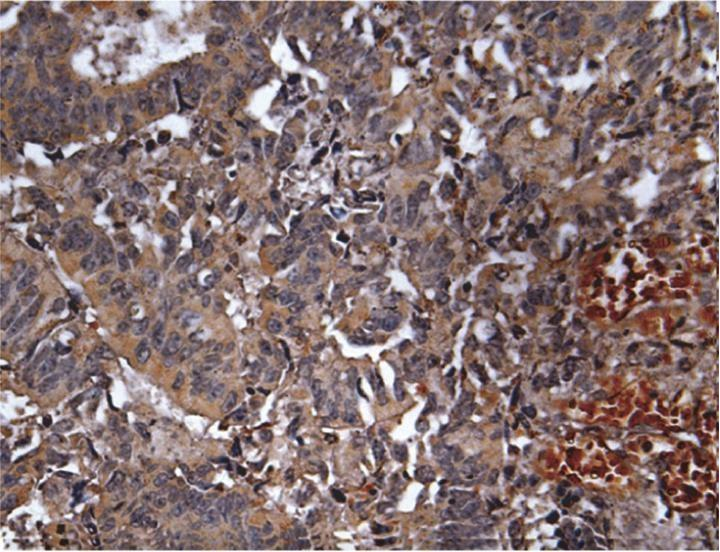
(F)

Elevated expression of the AJUBA protein exhibits significant associations with key clinicopathological parameters in colorectal carcinoma. A retrospective analysis of 90 FFPE CRC specimens was conducted, encompassing seven cases at pathological stage I, 48 at stage II, 33 at stage III, and 2 at stage IV. Notably, IHC staining revealed pronounced AJUBA protein abundance in 50 of these specimens (55.56%), whereas 40 lesions (44.44%) displayed either faint or undetectable immunoreactivity (Table 1). Comparative assessment between neoplastic and juxtaposed nonneoplastic colonic mucosa demonstrated markedly higher AJUBA expression levels within malignant tissues, contrasting with minimal or absent staining in adjacent normal counterparts. Further quantitative analyses were implemented to systematically evaluate potential correlations between AJUBA protein abundance and established clinicopathological variables. As illustrated in Figure 9C–F, representative micrographs depict conspicuous differences in AJUBA immunolabeling intensity between primary CRC lesions and adjacent histologically normal tissues, with the latter exhibiting predominantly weak staining patterns. Subcellular localization studies confirmed predominant cytoplasmic distribution of the AJUBA protein within tumor cells.

Comprehensive analyses were conducted to explore the potential relationships between the expression levels of the AJUBA protein and a range of clinical characteristics, including gender, age, tumor (T) stage, metastasis (M) stage, tumor size, histological grade, and the extent of tumor infiltration. As shown in Table 1, no statistically significant correlations were found between AJUBA protein expression and factors such as the age of the patients, the size of the tumors, or the histological grade among those diagnosed with CRC. However, a notably strong association was identified between AJUBA expression and both the nodal (N) stage (*p* = 0.001) and the overall TNM staging system (*p* = 0.001).

### 3.8. AJUBA Knockdown Inhibits the Proliferation, Migration, Invasion, and Xenograft Tumor Growth of CRC Cells

Previous studies have demonstrated a notable increase in the expression levels of the AJUBA protein in CRC tissues. To investigate the functional significance of AJUBA in CRC, we designed two distinct siRNA sequences—specifically, siRNA‐AJUBA‐1 and siRNA‐AJUBA‐2—to selectively silence AJUBA expression. A scrambled siRNA sequence was used as a negative control to ensure that any observed effects were due to AJUBA knockdown and not nonspecific impacts of the transfection process. Each siRNA was introduced into CRC cell lines via RNA interference (RNAi), and the efficiency of AJUBA knockdown was evaluated using Western blot analysis, with results shown in Figure 10A.

Figure 10Suppression of AJUBA expression inhibits colorectal cancer cell proliferation, migration, invasion, and the growth of xenograft tumors. (A) Western blot analysis demonstrated effective downregulation of AJUBA protein levels. (B, C) A decline in cell proliferation was observed after AJUBA knockdown, as determined by CCK‐8 and colony formation assays. (D, E) Transwell migration and invasion assays showed that silencing AJUBA significantly reduced the migratory and invasive capabilities of the cells. (F,G) Representative images and growth curves of xenograft tumors are presented, showing reduced tumor development 15 days after treatment with si‐AJUBA‐1 compared to the control group (NC). (H) Tumor weight was measured and compared between the si‐AJUBA‐1 and NC groups on day 13 postinjection. (I) Immunohistochemical staining illustrated changes in the expression of Ki‐67 and AJUBA in xenograft tumor tissues. Representative photomicrographs are shown. Scale bars represent 20 μm. Statistical significance:  ^∗∗^ *p* < 0.01,  ^∗∗∗^ *p* < 0.001.

**Figure:**
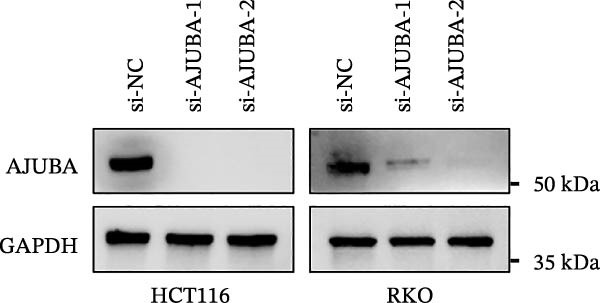
(A)

**Figure:**
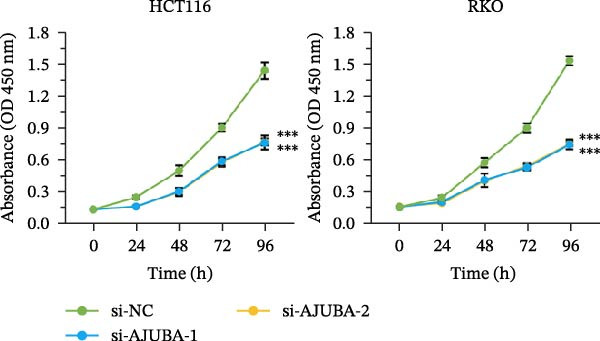
(B)

**Figure:**
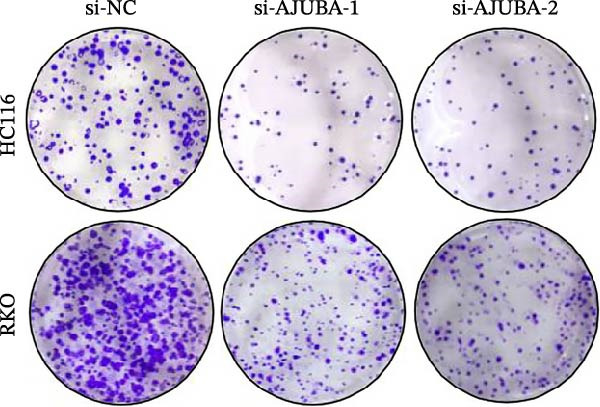
(C)

**Figure:**
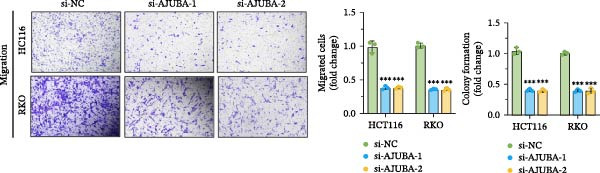
(D)

**Figure:**
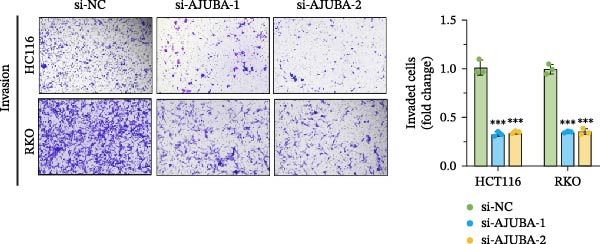
(E)

**Figure:**
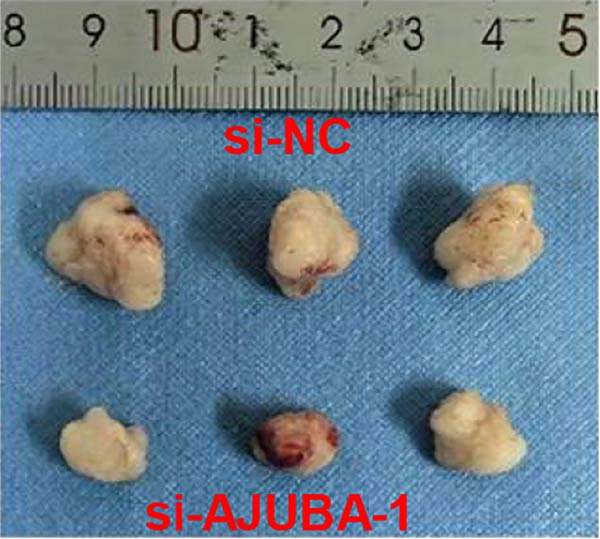
(F)

**Figure:**
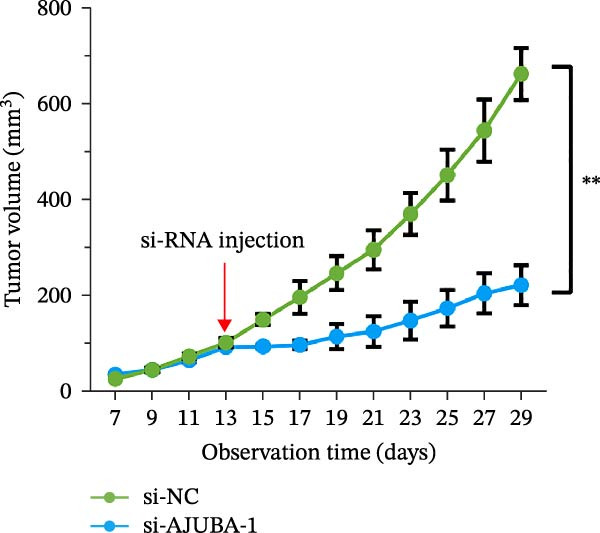
(G)

**Figure:**
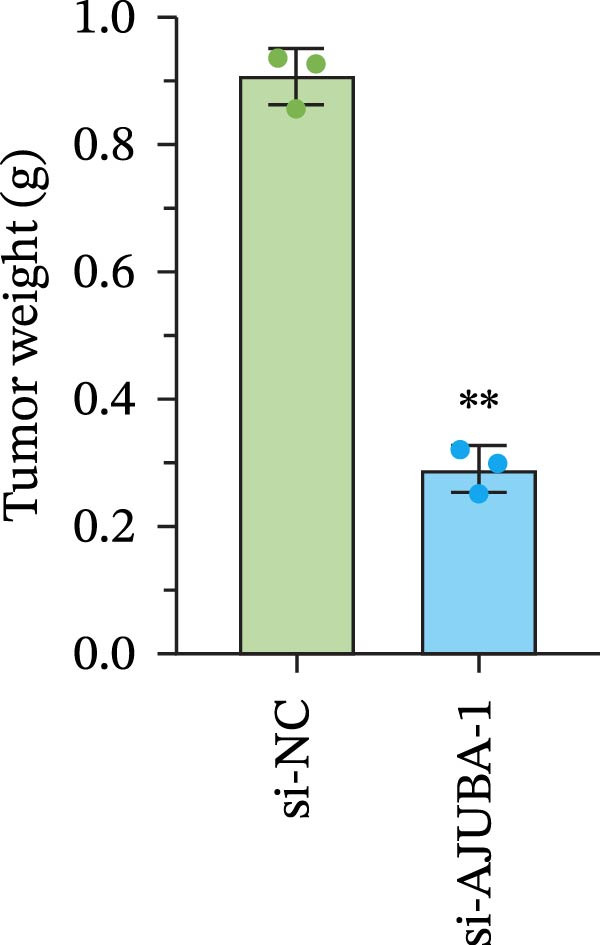
(H)

**Figure:**
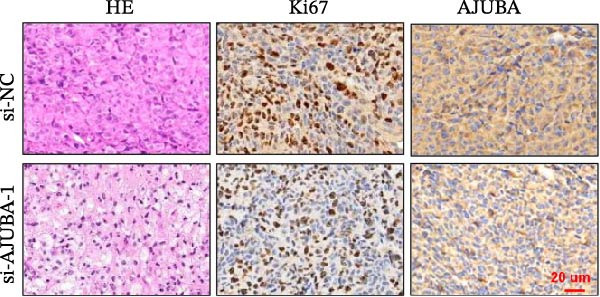
(I)

Following successful knockdown, functional assays were conducted to assess the biological roles of AJUBA. Cell proliferation was measured using both the CCK‐8 assay and colony formation assays, which revealed that silencing AJUBA markedly impaired the growth potential of HCT116 and RKO cell lines (Figure 10B,C). To further explore the involvement of AJUBA in cancer metastasis, Transwell migration and invasion assays were performed. These experiments demonstrated that reducing AJUBA expression significantly suppressed the migratory and invasive capabilities of CRC cells (Figure 10D,E).

To examine the in vivo relevance of these findings, we employed a xenograft tumor model in mice. Starting on Day 13 post‐cell injection, mice were administered either negative control siRNA or AJUBA‐targeting siRNA at 2‐day intervals, and were euthanized on Day 29. Tumor growth analysis showed that tumors derived from HCT116 cells with reduced AJUBA expression grew at a considerably slower rate compared to those formed from cells treated with the negative control siRNA (Figure 10F,G). Additionally, tumors treated with si‐AJUBA‐1 exhibited a significant reduction in weight when compared to the negative control group (Figure 10H). IHC staining of the excised tumors further confirmed that AJUBA knockdown led to decreased expression of both AJUBA and the proliferation marker Ki67, relative to the negative control group (Figure 10I).

In summary, the collective data strongly suggest that AJUBA plays a critical role in promoting cell proliferation, facilitating metastatic behavior, and driving overall tumorigenic progression in CRC.

### 3.9. Association Between AJUBA Expression and Patient Survival

The assessment of survival outcomes revealed a statistically significant negative correlation between the expression level of the AJUBA protein and OS in patients diagnosed with CRC (*p* = 0.001, Figure 11A). Additional multivariate Cox regression analysis further identified AJUBA expression, in addition to lymph node (N) stage and distant metastasis (M) stage, as independent factors predictive of OS (Table 2).

Figure 11Univariate survival analysis using Kaplan–Meier method and log‐rank test for significance. (A) Overall survival (OS) analysis comparing patients with high AJUBA protein expression versus those with low AJUBA levels in the entire patient cohort. (B) Survival analysis focused on node‐negative (N0) patients, differentiating outcomes based on high and low AJUBA expression. (C) Survival outcome assessment in node‐positive (N1−2) cases, examining survival differences between high and low AJUBA expression. (D) Overall survival assessment in early‐stage colorectal cancer (TNM Stage I‐II), comparing survival courses between high and low AJUBA expression subgroups. (E) Overall survival evaluation in advanced‐stage colorectal cancer (Stage III–IV), comparing survival trends between patients with high and low AJUBA expression. (F) Survival evaluation in patients with tumors smaller than 5 cm, investigating survival variations between high and low AJUBA expression groups. (G) Overall survival analysis for tumors larger than 5 cm, assessing survival results based on varying AJUBA expression levels. (H) Survival analysis in well‐differentiated colorectal cancer (Grades 1–2), comparing overall survival between patients with high and low AJUBA expression. (I) Survival outcome assessment in poorly differentiated colorectal cancer (Grade 3), evaluating survival differences between high and low AJUBA expression groups.

**Figure:**
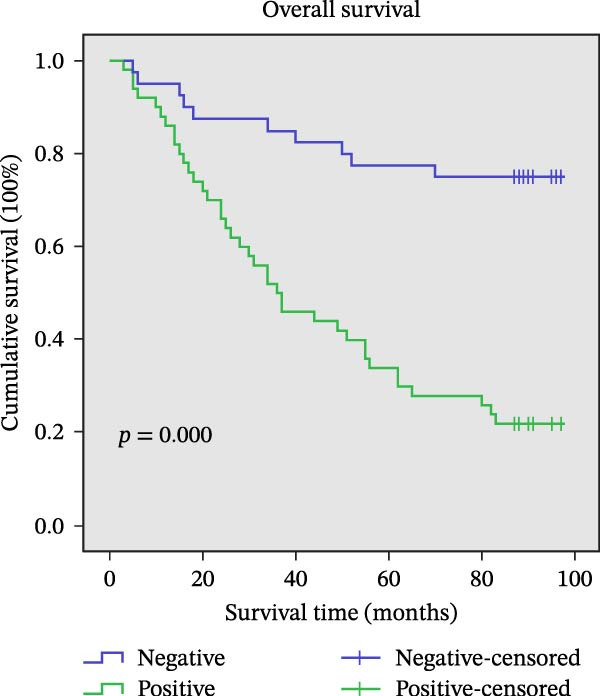
(A)

**Figure:**
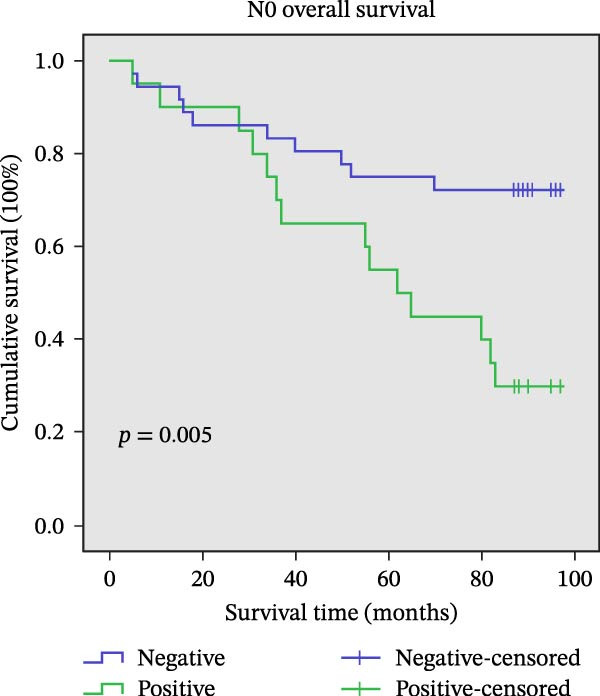
(B)

**Figure:**
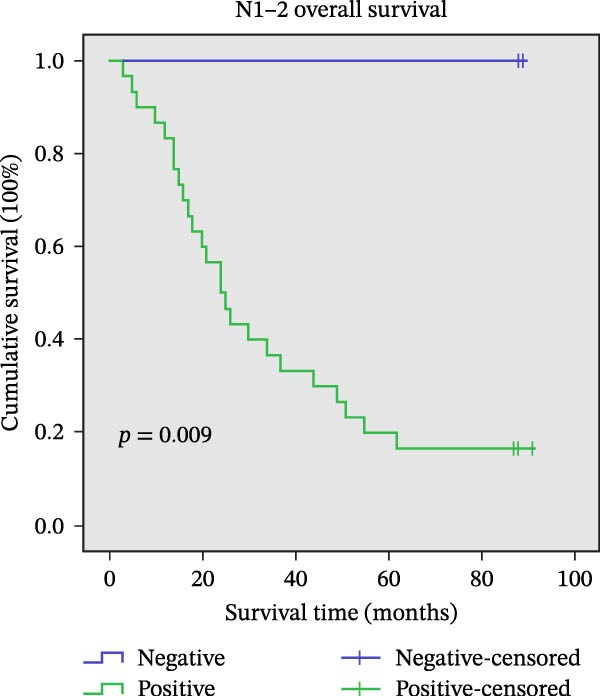
(C)

**Figure:**
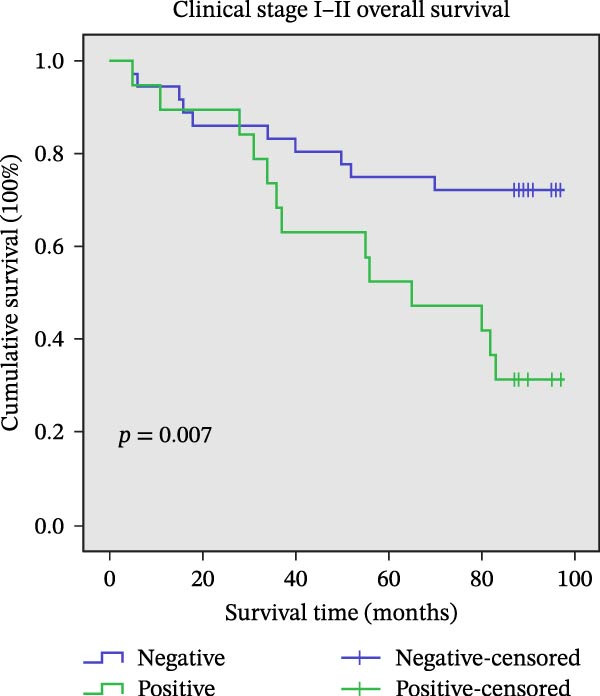
(D)

**Figure:**
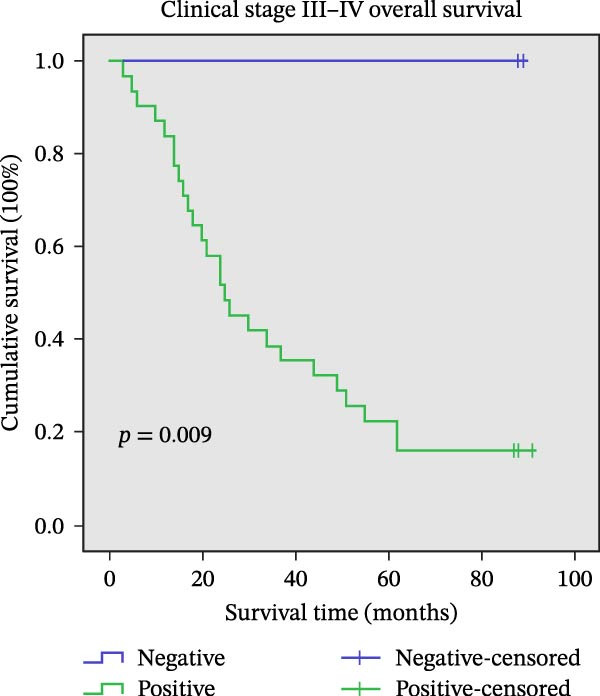
(E)

**Figure:**
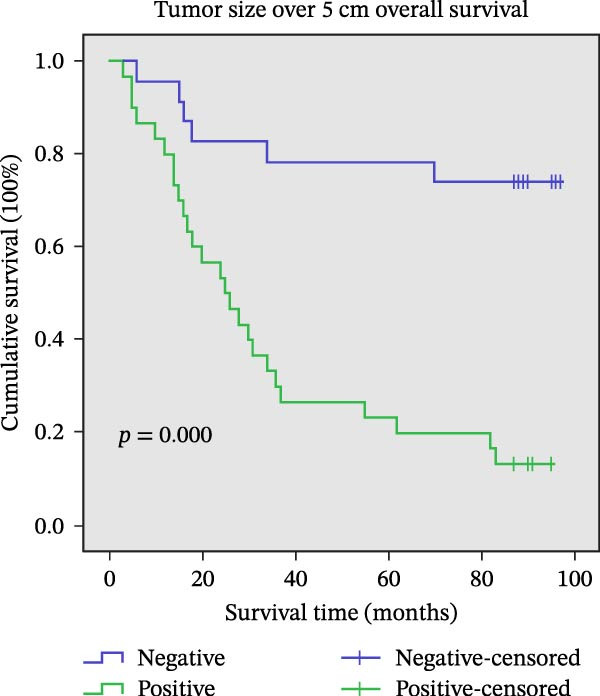
(F)

**Figure:**
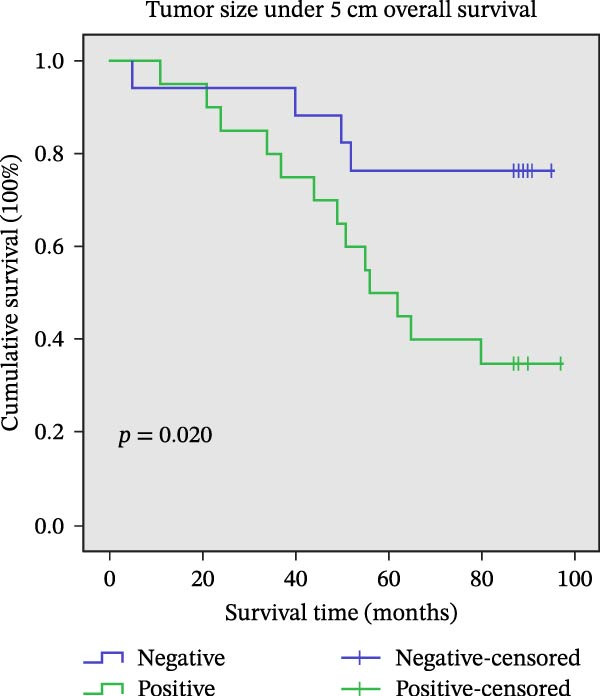
(G)

**Figure:**
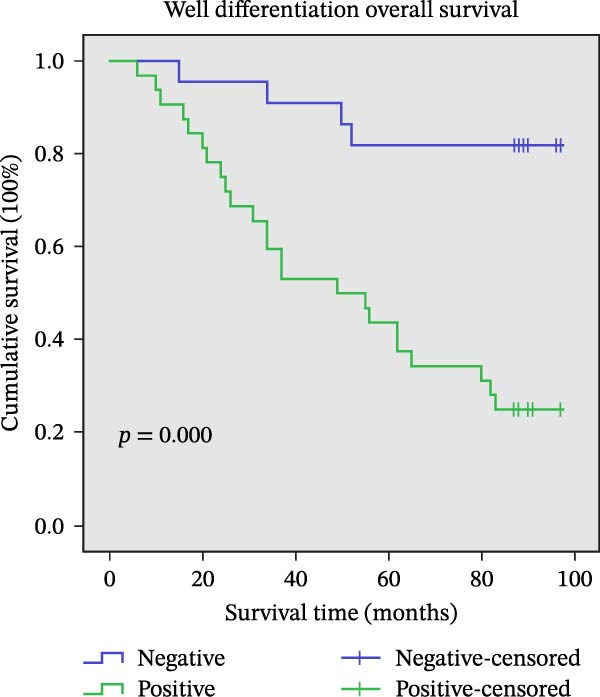
(H)

**Figure:**
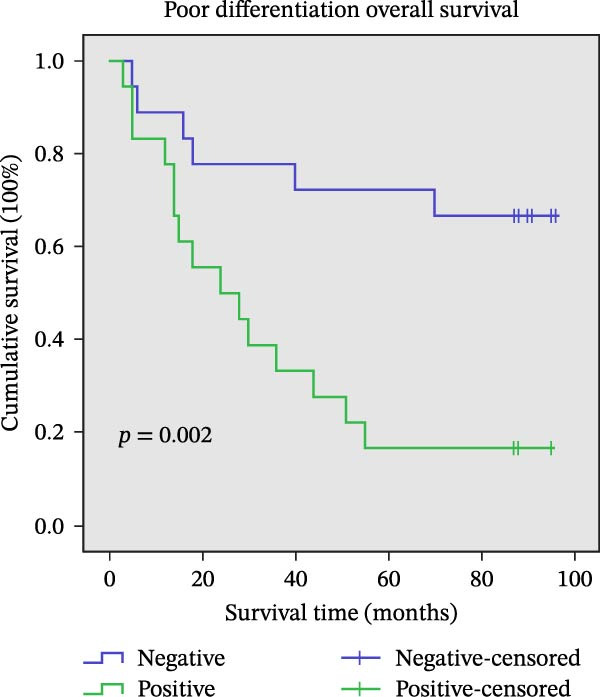
(I)

**Table 2:** Cox‐regression analysis of various prognostic parameters in patients for all patients.

Factor	Univariate		Multivariate	
	HR (95%CI)	*p*‐Value	HR (95%CI)	*p*‐Value
N Stage				
0	Reference	0.000	Reference	0.004
1	2.036 (1.079–3.842)	0.028	1.172 (0.588–2.337)	
2	7.496 (3.283–17.114)	0.000	4.212 (1.770–10.026)	
M Stage				
0	Reference			
1	11.072 (2.509–48.856)	0.001	9.221 (1.934–43.956)	0.005
Infiltration				
0	Reference		—	—
1	4.580 (1.355–15.486)	0.014		
AJUBA expression				
Negative	Reference			
Positive	4.809 (2.385–9.695)	0.000	3.758 (1.762–8.014)	0.001
TNM stage				—
1	Reference	0.000		
2	1.096 (0.325–3.689)	0.883		
3	2.784 (0.835–9.283)	0.096		
4	18.812 (2.941–120.317)	0.002	—	

We further explored the prognostic significance of AJUBA expression across various patient subgroups, including those stratified by lymph node involvement (N stage), overall tumor stage (TNM classification), tumor size, and histological grade. Subgroup analyses demonstrated that elevated AJUBA expression was consistently associated with poorer OS. This relationship was observed both in patients with no lymph node metastasis (N0 group, Figure 11B, log‐rank test, *p* = 0.005) and in those with regional lymph node involvement (N1–2 group, Figure 11C, log‐rank test, *p* = 0.009).

In early‐stage CRC (Stage I–II), higher AJUBA levels were markedly linked to reduced survival (Figure 11D, log‐rank test, *p* = 0.007), and a similar trend was evident in patients with more advanced disease (Stage III–IV, Figure 11E, log‐rank test, *p* = 0.009). When the analysis was performed based on tumor size, AJUBA expression retained its prognostic value in both large tumors (greater than 5 cm, Figure 11F, log‐rank test, *p* = 0.001) and smaller tumors (less than 5 cm, Figure 11G, log‐rank test, *p* = 0.020).

Furthermore, significant associations between AJUBA expression and survival outcomes were also noted in tumors classified by histological differentiation. Specifically, in well‐differentiated tumors (Grades 1–2, Figure 11H, log‐rank test, *p* = 0.002) and poorly differentiated tumors (Grade 3, Figure 11I, log‐rank test, *p* = 0.001), increased AJUBA expression was correlated with worse OS.

## 4. Discussion

The EMT represents a dynamic and reversible process that promotes intratumoral heterogeneity, metastasis, and treatment resistance in CRC [35, 36]. While numerous EMT‐related gene signatures have been discovered, their clinical applicability remains limited by tumor heterogeneity and insufficient biomarkers for prognostic stratification [3, 37]. In this study, we implemented a comprehensive multi‐omics framework combining bulk RNA‐seq, single‐cell transcriptomics, and machine learning to systematically evaluate EMT‐associated genes with biological and clinical importance. Within multiple independent cohorts, AJUBA was consistently identified as the most significantly upregulated gene exhibiting poor prognostic associations among leading candidates, motivating further analysis of its functional contributions to CRC development.

AJUBA, a constituent of the LIM protein family, contains three consecutive LIM domains at its C‐terminus and diverse proline‐rich N‐terminal regions referred to as the preLIM domain [8, 38, 39]. The LIM domain, first identified in *Caenorhabditis elegans* Mec‐3, rat Isl‐1, and *C. elegans* Lin‐11, consists of two zinc‐finger motifs, forming the basis for its acronym [40, 41]. Members of the LIM protein family are categorized according to the quantity of LIM domains, the degree of sequence conservation, and their structural organization within proteins. AJUBA is classified within the Zyxin/AJUBA subfamily alongside Zyxin, Limd1, Lpp, Trip6, and Wtip, each contributing critically to integrin‐mediated and cell–cell adhesion complexes [42]. No evidence supports direct DNA binding by the AJUBA/Zyxin subfamily; instead, LIM domains primarily serve as platforms for protein–protein interactions, operating across cellular compartments to enable diverse biological functions [8, 10, 43, 44]. A unique nuclear export signal (NES) is situated within AJUBA’s preLIM domain; deletion of this NES or the entire preLIM region leads to nuclear accumulation of AJUBA [8, 45]. Furthermore, the preLIM domain participates in protein interactions, exemplified by its association with HDACs and Prmt5 [46, 47]. Owing to pronounced structural and functional conservation across AJUBA family proteins, multiple studies indicate that these molecules can engage common interaction partners or form analogous complexes, thereby regulating similar cellular processes [43, 48, 49]. Nonetheless, each protein retains unique functions. For instance, Witzel et al. [50] reported that JUB exhibited the strongest binding to Isl1 among the proteins tested, while LIMD1 demonstrated the weakest affinity. Employing LAST2 as bait identified JUB as the exclusive binding partner within this family [51]. Proteins of the AJUBA family participate in a range of cellular functions such as transcriptional regulation, proliferation, adhesion, migration, and cell division [44]. Although detailed molecular mechanisms are not fully elucidated, it is established that these processes are governed by precise localization of signaling molecules—encompassing binding events, intermolecular interactions, and the formation of functional complexes through tethering mechanisms [52, 53]. AJUBA differs from other family members in several respects. First, unlike all other proteins in this group, AJUBA is absent from focal adhesion sites [8, 54–56]. Consistent with its familial characteristics, AJUBA localizes to regions of cell–cell contact [54–57]. In settings where intercellular contacts are missing, AJUBA displays a diffuse cytoplasmic pattern. These observations imply that the recruitment of AJUBA to the cell surface constitutes a regulated, active process rather than a passive occurrence.

AJUBA functions in the regulation of diverse cellular activities, such as cell fate determination [45, 58], oocyte meiotic maturation [8], transcriptional repression [10, 43, 46, 47, 59], DNA damage response [60], actin cytoskeleton regulation [9], intercellular adhesion, [9] as well as cell migration and invasion [61].

This functional versatility arises from its ability to associate with multiple signaling and structural proteins—for instance, Grb2 [8], 14‐3‐3 proteins [62], and PIPKI‐a [63], and to modulate essential signaling cascades, including the Wnt [64] and Rac pathways [65]. Although AJUBA acts as an actin‐binding protein [9], the specific mechanisms through which it participates in actin‐dependent cytoskeletal reorganization are not yet fully clarified. Recruitment of AJUBA to cellular junctions occurs via direct binding of its LIM domains to α‐catenin, and its preLIM domain engages directly with filamentous actin (F‐actin) [9]. Therefore, AJUBA potentially strengthens junctional integrity either by reorganizing F‐actin at adhesion sites or by linking cadherin complexes to the cytoskeletal framework.

AJUBA functions as a key regulatory component in the mitotic apparatus, facilitating Aurora‐A kinase activation to permit CDK1–cyclin B complex formation at centrosomal areas, thereby driving mitotic progression [66]. During mitosis, AJUBA engages in direct binding with Lats2 at centrosomes and aids in preserving spindle integrity [51]. Furthermore, AJUBA associates with microtubules and assists the metaphase‐to‐anaphase shift by interacting with Aurora‐B and BubR1 at kinetochores [67]. Together, these observations emphasize the vital contribution of AJUBA to mitotic control and propose that perturbation of its functions during division may yield context‐dependent effects, either oncogenic or tumor‐suppressive.

The presence of AJUBA mutations in human cancers, including cutaneous squamous cell carcinoma [16], head and neck squamous cell carcinomas [11], and esophageal squamous cell carcinoma [12, 13], offers growing support for its participation in tumorigenesis. Notably, the involvement of AJUBA in cancer development continues to be actively contested [14, 61]. While some investigations propose that AJUBA stimulates cell proliferation, other reports reveal it imposes growth‐restraining influences on malignant mesothelioma cells [14], illustrating its context‐specific activities in cancer. This diversity in function complicates the precise definition of AJUBA’s biological contributions to tumor formation.

Emerging findings suggest that the EMT represents a spectrum of states rather than a binary switch, encompassing intermediate phases that may be governed by distinct molecular mechanisms [36]. Single‐cell analyses have revealed considerable heterogeneity in AJUBA expression within tumor populations, with pronounced enrichment in a subpopulation of epithelial cells that coexpress established EMT markers—such as components of the VEGF, NOTCH, and PI3K‐AKT signaling pathways. These observations imply that AJUBA might act as a regulator initiating EMT rather than serving as an indicator of full mesenchymal conversion. Future lineage‐tracing studies will be crucial to determine whether AJUBA‐high cells represent a transient intermediate state with increased metastatic potential.

Notably, AJUBA‐positive epithelial cells showed strengthened communicative interactions with endothelial cells via VEGF signaling, indicating a possible role for AJUBA in promoting angiogenesis during EMT in tumor progression. These data are consistent with recent work connecting EMT activation to vascular co‐option and the formation of pre‐metastatic niches [68–70]. While it remains unclear whether AJUBA‐enhanced VEGF signaling is a cause or an effect of EMT, our findings suggest that AJUBA inhibition could simultaneously suppress EMT and disrupt tumor‐stromal communication, offering a potential dual‐therapeutic approach for metastatic CRC.

In summary, our results identify AJUBA as an independent prognostic marker, although the precise molecular mechanisms through which it regulates EMT are not yet fully understood. Preliminary experimental data demonstrate that depletion of AJUBA in CRC cells reduces migratory and invasive potential, suppresses VEGF secretion, and decreases levels of key EMT transcription factors, including Snail and Twist1 (unpublished data). These findings are consistent with our in silico predictions and reinforce the hypothesis that AJUBA modulates EMT via transcriptional mechanisms. Additional studies using CRISPR‐based gene editing in patient‐derived organoids are needed to determine if AJUBA may function not only as a biomarker but also as a candidate therapeutic target in CRC.

As demonstrated in this study, heightened AJUBA expression is linked to adverse clinical outcomes in CRC. Our data reveal substantially elevated AJUBA mRNA and protein levels in tumor specimens relative to matched nonneoplastic colorectal tissues. These results confirm AJUBA’s potential as a diagnostic biomarker capable of improving accuracy in CRC detection. Nonetheless, the pathological functions of AJUBA in human cancers are not yet fully elucidated. Essential cellular mechanisms—such as migration, adhesion, and morphological adaptability—play critical roles in tumor dissemination and metastatic progression, implying that AJUBA may act as a modulator of these processes. Further research will be necessary to delineate the specific signaling pathways through which AJUBA contributes to colorectal oncogenesis.

Additional analyses were conducted to examine associations between AJUBA expression and clinicopathological parameters in CRC patients. Our findings demonstrated that AJUBA levels were directly correlated with N stage (denoting lymph node metastasis) and TNM stage (an integrated staging system encompassing tumor invasion, nodal status, and metastatic spread). These observations strengthen the concept that increased AJUBA expression corresponds to more advanced disease, particularly in contexts of lymph node involvement and overall tumor progression. Conversely, no statistically significant relationships were detected between AJUBA expression and other clinical variables, including sex, age, T stage (degree of primary tumor invasion), M stage (presence of distant metastases), tumor size, histological grade, or local invasiveness.

Prior studies have documented conflicting results concerning AJUBA’s function in cancer development [20, 55]. To date, no published work has validated the prognostic significance of AJUBA specifically in CRC. Our multivariate analysis indicated that AJUBA expression functions as an autonomous predictor of OS among CRC patients (Table 2). These outcomes underscore the possibility that elevated AJUBA expression may operate as a prognostic biomarker to evaluate disease progression and patient survival. Translational exploitation of AJUBA will face druggability challenges. As a purely scaffolding LIM‐domain protein it lacks enzymatic pockets, and its oncogenic activity relies on multivalent protein–protein interactions (e.g., with α‐catenin, LATS2, and Snail) [9, 46, 51]. Consequently, conventional small‐molecule catalytic inhibitors are not applicable. Emerging approaches include stapled peptides that competitively disrupt the AJUBA–α‐catenin interface [71] or mini‐protein binders that occlude the LIM‐domain binding grooves [72]; however, these strategies are still preclinical and will require optimization for in vivo stability and tumor‐specific delivery. Until such hurdles are overcome, AJUBA’s most immediate clinical value remains its robust prognostic power rather than direct therapeutic targeting.

## 5. Conclusions

In conclusion, our integrated approach—combining transcriptomic data from multiple cohorts, single‐cell sequencing profiles, and machine learning systems—allowed the systematic identification of AJUBA as a pivotal gene associated with EMT in CRC. AJUBA is significantly elevated in malignant tissues and identifies a subset of epithelial cells displaying active EMT along with proangiogenic signaling activation. Its expression represents an independent predictor of reduced OS, irrespective of nodal status, TNM stage, or tumor size. From a functional perspective, AJUBA may act as an initiator of EMT and an enabler of tumor‐stromal crosstalk, rather than a bystander molecule. These results position AJUBA as a multifunctional biomarker that improves risk stratification and guides therapeutic strategies targeting EMT‐mediated metastasis. Subsequent validation efforts and in‐depth mechanistic studies employing patient‐derived models will be critical to advancing AJUBA toward clinical application.

## Author Contributions

This research project was originally conceptualized and planned by Cuijie Shao, Wenhui Shen, and Biji Zou. The hands‐on experimental procedures and the drafting of the initial manuscript were carried out by Xiaojun Zhang and Minghui Cui. Xiaoqian Liao and Yuhan Xiong were responsible for conducting the statistical evaluations. In addition to his experimental contributions, Xiaojun Zhang also played a role in further manuscript development and data interpretation.

## Funding

The financial support for this study was supplied by several institutions, including the Shandong Natural Science Foundation of China (Grant ZR2017MH125), the Binzhou Medical University Program (Grant BY2015KJ01), the Shandong Traditional Chinese Medicine Science and Technology Development Plan (Grant 2019‐0515), the National Key Clinical Discipline Construction Project, the Henan Provincial Science and Technology Research Project (Grant 242102311206), and the Joint Construction Project of Medical Science and Technology in Henan Province (Grant LHGJ20240102).

## Disclosure

Every author participated in the thorough review, revision, and final approval of the completed manuscript.

## Ethics Statement

The Ethics Committee at the Binzhou Medical College Hospital approved this study (Ethics Approval Number: 2022 [LW‐50]).

## Consent

All the patients provided written informed consent for the use of clinical specimens for medical research.

## Conflicts of Interest

The authors declare no conflicts of interest.

## Data Availability

The data and material of this study are included in this published article.
